# Osmotic stress induces the phosphorylation of WNK4 Ser575 via the p38MAPK-MK pathway

**DOI:** 10.1038/srep18710

**Published:** 2016-01-06

**Authors:** Junichi Maruyama, Yumie Kobayashi, Tsuyoshi Umeda, Alain Vandewalle, Kohsuke Takeda, Hidenori Ichijo, Isao Naguro

**Affiliations:** 1 Laboratory of Cell Signaling, Graduate School of Pharmaceutical Sciences, The University of Tokyo, 7-3-1 Hongo, Bunkyo-ku, Tokyo 113-0033, Japan; 2 Department of Medical Biochemistry, Graduate School of Medical and Dental Sciences, Tokyo Medical and Dental University, 1-5-45 Yushima, Bunkyo-ku, Tokyo 113-8510, Japan; 3 Centre de Recherche sur l’Inflammation, UMRS 1149, Université Paris 7, site Bichat, 75018, Paris, France; 4 Cell Regulation, Graduate School of Biomedical Sciences, Nagasaki University, 1-14 Bunkyo-machi, Nagasaki 852-8521, Japan

## Abstract

The With No lysine [K] (WNK)-Ste20-related proline/alanine-rich kinase (SPAK)/oxidative stress-responsive kinase 1 (OSR1) pathway has been reported to be a crucial signaling pathway for triggering pseudohypoaldosteronism type II (PHAII), an autosomal dominant hereditary disease that is characterized by hypertension. However, the molecular mechanism(s) by which the WNK-SPAK/OSR1 pathway is regulated remain unclear. In this report, we identified WNK4 as an interacting partner of a recently identified MAP3K, apoptosis signal-regulating kinase 3 (ASK3). We found that WNK4 is phosphorylated in an ASK3 kinase activity-dependent manner. By exploring the ASK3-dependent phosphorylation sites, we identified Ser575 as a novel phosphorylation site in WNK4 by LC-MS/MS analysis. ASK3-dependent WNK4 Ser575 phosphorylation was mediated by the p38MAPK-MAPK-activated protein kinase (MK) pathway. Osmotic stress, as well as hypotonic low-chloride stimulation, increased WNK4 Ser575 phosphorylation via the p38MAPK-MK pathway. ASK3 was required for the p38MAPK activation induced by hypotonic stimulation but was not required for that induced by hypertonic stimulation or hypotonic low-chloride stimulation. Our results suggest that the p38MAPK-MK pathway might regulate WNK4 in an osmotic stress-dependent manner but its upstream regulators might be divergent depending on the types of osmotic stimuli.

With no lysine [K] (WNK) kinases, which consist of WNK1, WNK2, WNK3 and WNK4, comprise a unique kinase family characterized by their irregular positioning of the lysine residue that is required for ATP binding^1^,^2^. Among them, WNK1 and WNK4 were reported to be responsible for pseudohypoaldosteronism type II (PHAII), which is an autosomal dominant hereditary disease characterized by hypertension, hyperkalemia and metabolic acidosis^3^. As this hypertension is effectively cured by treatment with thiazide diuretics, hyperactivation of the Na-Cl cotransporter (NCC), the target of thiazide diuretics, is thought to be the main cause of PHAII hypertensive symptoms. In 2001, Wilson *et al.* identified *PRKWNK1* and *PRKWNK4* as the genes responsible for PHAII by positional cloning^3^. The mutant *PRKWNK1* gene was found to lack intron 1, which ultimately leads to the overexpression of the wild-type WNK1 protein at the distal convoluted tubule, as well as ectopic expression of the kidney-specific WNK1 isoform^1^,^3^,^4^. The mutant *PRKWNK4* gene was found to incorporate the missense mutations E562K, D564A, Q565E and R1185C^1^,^3^. In another study, Ste20-related proline/alanine-rich kinase (SPAK) and oxidative stress-responsive kinase 1 (OSR1) were found to be hyperactivated in the kidneys of WNK4 D561A knock-in mice^5^,^6^. SPAK and OSR1 have also been identified as direct upstream kinases of NCC^7^,^8^. From these observations, the hyperactivation of the WNK-SPAK/OSR1-NCC pathway is gaining attention as one of the main molecular mechanisms underlying hypertension in PHAII patients^1^,^9^,^10^. On the other hand, WNK4 hypomorphic mice and SPAK kinase-inactive mutant knock-in mice have been reported to exhibit similar hypotensive phenotypes^11^,^12^. The angiotensin II-dependent activation of the SPAK-NCC pathway has also been reported to be impaired in WNK4 knockout mice^13^. Importantly, dietary salt regulates the activity of the WNK-SPAK/OSR1-NCC pathway through the regulation of aldosterone, suggesting that the WNK-SPAK/OSR1 pathway is also involved in the physiological regulation of blood pressure^14^.

Three of the four PHAII mutations in WNK4 lie within the narrow acidic region (E562K, D564A and Q565E in human WNK4), which suggests the importance of this region in the molecular mechanism of PHAII. Recent studies have clarified that the acidic region forms a binding site for Kelch-like protein 3 (KLHL3), which is a substrate adaptor protein of the Cullin3 E3 ligase complex^15^. All WNK4 mutations in the acidic region disrupt the interaction with the E3 ligase complex, which impairs the degradation of WNK4 by the ubiquitin-proteasome system^16^,^17^,^18^. The resulting increase in WNK4 protein levels contributes to the activation of the SPAK/OSR1-NCC pathway and PHAII pathogenesis^19^.

Apoptosis signal-regulating kinases (ASKs), which are MAP3Ks of the stress-activated MAPK pathway, respond to various stressors and activate the JNK and p38MAPK pathways^20^,^21^. Recently, we reported that a newly identified member of the ASK family, ASK3, which is highly related to ASK1, negatively regulates WNK1 activity and that ASK3 knockout mice show a moderate hypertensive phenotype when given a high-salt diet^22^,^23^. Taken together, we hypothesize that ASK3 maintains blood pressure by modulating the activity of the WNK1-SPAK/OSR1 pathway. However, the precise molecular mechanism by which ASK3 regulates the WNK1-SPAK/OSR1 pathway is largely unknown. Moreover, the involvement of ASK3 in the regulation of other WNK kinases still remains elusive.

In the present study, we identified WNK4 as an ASK3-interacting partner, and found that ASK3 induced the phosphorylation of WNK4 at Ser575. The Ser575 phosphorylation was also induced by the osmotic stress via the p38MAPK-MK pathway, and ASK3 was required for the hypotonic stress-induced WNK4 Ser575 phosphorylation. From these results, we propose a potential model in which WNK4 might be regulated by the stress-activated MAPK signaling pathway.

## Results

### WNK4 phosphorylation is increased by the co-expression of ASK3

**Figure 1:**
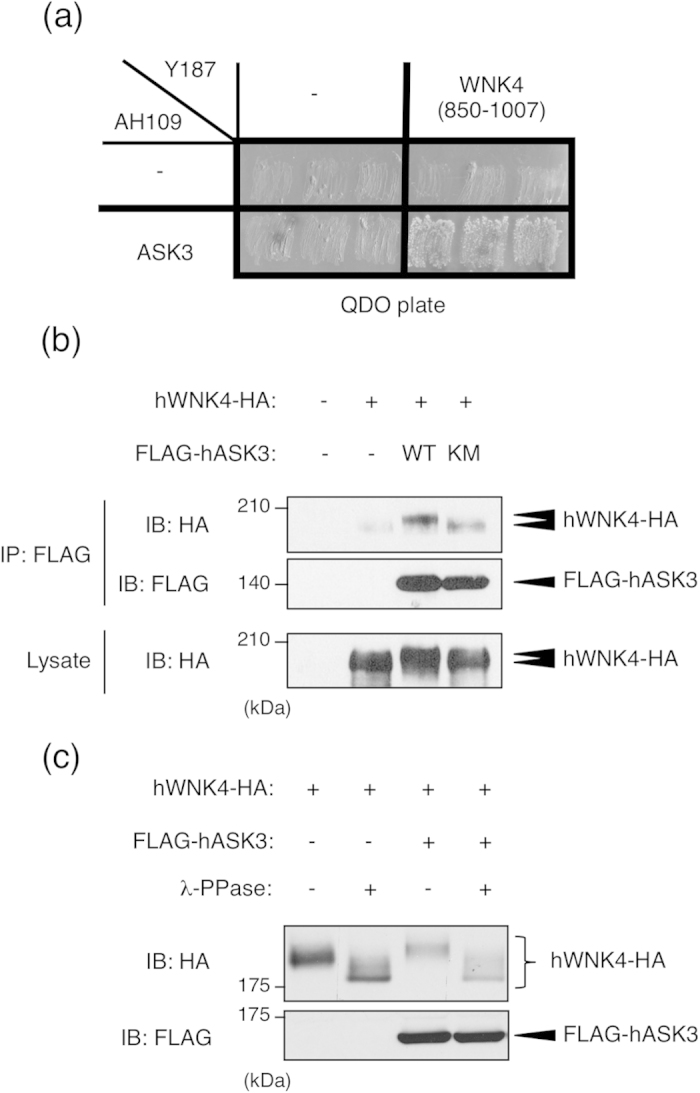
ASK3 binds to WNK4 and induces WNK4 phosphorylation in cells. (**a**) Interaction between ASK3 and WNK4 in yeast. Yeast strain Y187 containing a plasmid encoding human WNK4 (850–1007) fused to the GAL4 transcriptional activation domain was mated with the yeast strain AH109 containing a plasmid encoding human ASK3-KM fused to the GAL4 DNA-binding domain. The mated yeast was able to survive on restriction medium (QDO). (**b**) Interaction between ASK3 and WNK4 in mammalian cells. HEK293A cells were transiently transfected with FLAG-tagged human ASK3 (FLAG-hASK3) wild type or human ASK3 kinase-inactive mutant (hASK3-KM) and HA-tagged human WNK4 (hWNK4-HA). Cells were lysed at 48 h after transfection. Lysates were immunoprecipitated with anti-FLAG antibody beads and subjected to immunoblotting (IB). Co-immunoprecipitated WNK4 was detected only in the co-transfected cells. Full-length blots are presented in Supplementary Fig. S5a. (**c**) The ASK3-dependent mobility shift of WNK4 band is due to phosphorylation. HEK293A cells were transiently transfected with FLAG-hASK3 and hWNK4-HA. Cells were lysed at 48 h after transfection. Lysates were divided, and a portion was treated with λ-phosphatase (λ-PPase) for 30 min at 30 °C. The reaction was terminated by the addition of SDS sample buffer, and samples were subjected to IB. Bands corresponding to both WNK4 that was expressed alone and WNK4 that was co-expressed with ASK3 were shifted lower and ran at the same position. Full-length blots are presented in Supplementary Fig. S5b.

Recently, we characterized ASK3 as a novel MAP3K protein kinase and found through immunohistochemical analysis that ASK3 protein was highly expressed in the renal tubules of the kidney^23^. To examine the physiological role of ASK3 in the kidney, we screened ASK3-interacting proteins using the yeast two-hybrid system. Using this approach, we identified WNK4 as an ASK3-interacting protein (Fig. 1a). The interaction between ASK3 and WNK4 was also confirmed in mammalian cells by co-immunoprecipitation analysis (Fig. 1b). This interaction was also observed when ASK3-K681M mutant, a kinase activity-deficient ASK3 mutant, was used. Osmotic stress, which affects the activity of ASK3, did not significantly affect this interaction (see Supplementary Fig. S1 online). Interestingly, the band corresponding to WNK4, especially in the IP fraction, was shifted higher in the presence of ASK3-WT co-expression than when WNK4 was expressed alone (Fig. 1b). This band shift was not observed when WNK4 was co-expressed with ASK3-K681M. The band corresponding to transfected WNK4 was shifted lower by λ phosphatase (λ-PPase) treatment, indicating that overexpressed WNK4 undergoes basal phosphorylation (Fig. 1c). Similarly, the WNK4 band, which was slightly shifted-up in the presence of ASK3, was shifted lower by λ-PPase treatment and ran at the same position as WNK4 alone did (Fig. 1c). These results suggested that the ASK3-dependent mobility shift of WNK4 represents the phosphorylation of WNK4 by the ASK3-dependent signaling pathway.

### WNK4 Ser575 is the ASK3-dependent phosphorylation site

**Figure 2:**
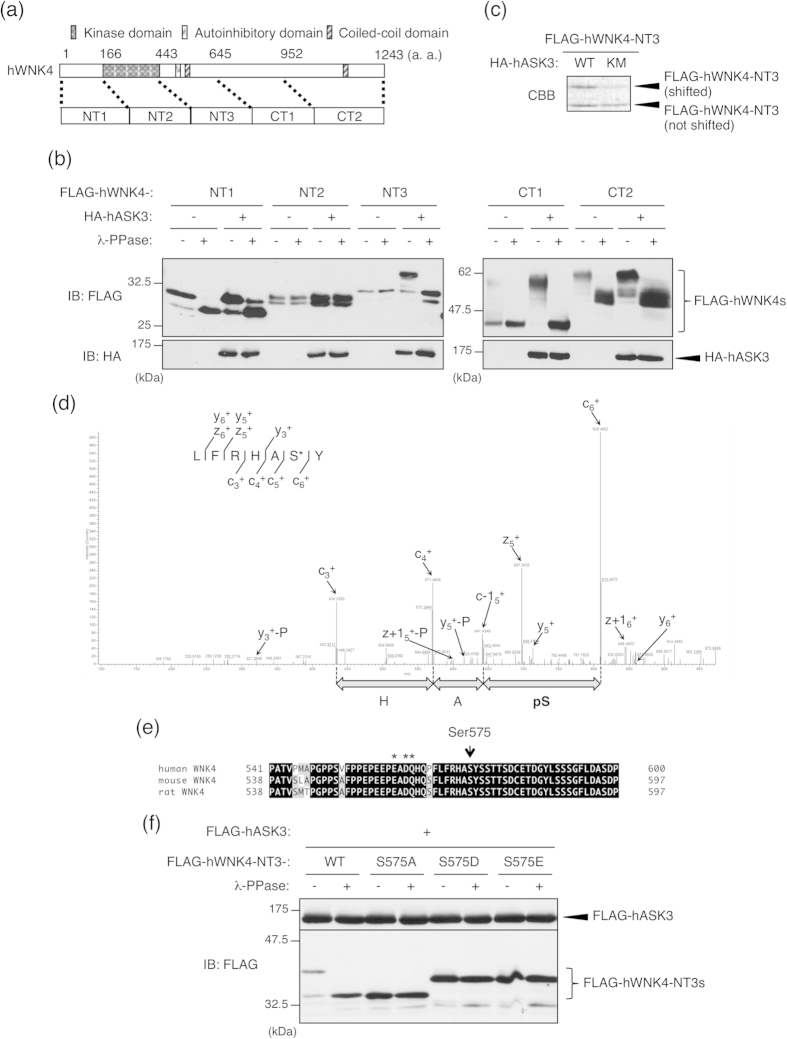
WNK4 Ser575 is an ASK3-dependent phosphorylation site. (**a**) A schematic view of WNK4. We constructed five FLAG-tagged WNK4 fragments (NT1/2/3, CT1/2) as indicated. (**b**) Mobility shift assay to narrow down the region containing ASK3-dependent phosphorylation sites. HEK293A cells were transiently transfected with HA-hASK3 and FLAG-tagged WNK4 fragments. Lysates were divided, and a portion was treated with λ-PPase for 30 min at 30 °C. The reaction was terminated by the addition of SDS sample buffer, and samples were subjected to IB. As a result, bands corresponding to fragments NT3 and CT1 were shifted higher following co-expression of ASK3, and this shift was abolished by λ-PPase treatment. Full-length blots are presented in Supplementary Fig. S6a. (**c**) HEK293A cells were transiently transfected with FLAG-hWNK4-NT3 and HA-hASK3-WT/KM. Cells were lysed at 48 h after transfection, and FLAG-hWNK4-NT3 proteins were immunoprecipitated with anti-FLAG antibody beads. Samples were subjected to SDS-PAGE and stained with CBB. (**d**) The ETD-MS/MS spectrum of peptide 138–143 from a chymotryptic digest of FLAG-hWNK4-NT3, localizing phosphorylation to Ser142 in the construct, which corresponds to Ser575 of human WNK4. Asterisk indicates identified phosphorylated residues. (**e**) Alignment of WNK4 orthologues. The Ser575 residue in human WNK4 (arrow) is conserved in murine WNK4 orthologues and is the nearest serine residue to the PHAII mutation sites (asterisks). (**f**) Ser575 phosphorylation is essential for the ASK3-dependent mobility shift of the NT3 fragment. HEK293A cells were transfected with FLAG-hASK3 and each FLAG-tagged hWNK4-NT3 mutants (WT, S575A, S575D and S575E). Lysates were divided and a portion was treated with λ-PPase for 30 min at 30 °C. The reaction was terminated by the addition of SDS sample buffer, and samples were subjected to IB. Full-length blots are presented in Supplementary Fig. S6b.

To narrow down the region containing the ASK3-dependent phosphorylation site(s), we constructed five WNK4 fragments, NT1 (1–166), NT2 (167–443), NT3 (444–645), CT1 (646–952), and CT2 (953–1243), and examined the fragment(s) that exhibited the ASK3-dependent mobility shift (Fig. 2a). Fragments NT3 and CT1 exhibited a mobility shift that was triggered by ASK3-dependent phosphorylation (Fig. 2b). The NT1 and CT2 fragments exhibited a phosphorylation-dependent mobility shift without ASK3 co-expression (Fig. 2b), which is consistent with the basal phosphorylation of WNK4 shown in Fig. 1c. In addition, we examined the interaction profile between ASK3 and WNK4 fragments (see Supplementary Fig. S2 online). ASK3 interacts majorly with NT2 and NT3 fragments and weakly with NT1 and CT1 fragments. From this data and Fig. 2b, it is suggested that ASK3 interacts with WNK4 through the region spanning the NT2 and NT3 fragments and induces the phosphorylation of the WNK4 NT3-CT1 region.

To identify the ASK3-dependent phosphorylation site(s), we then performed a LC-ETD-MS/MS analysis. NT3 fragments were co-expressed with ASK3-WT or ASK3-K681M mutant, immunoprecipitated with anti-FLAG antibody and separated by SDS-PAGE. The gel was stained with coomassie brilliant blue (CBB), and the upper NT3 fragment that was shifted higher by ASK3-WT co-expression, as well as the lower NT3 fragment that was not shifted higher by ASK3-K681M co-expression, were excised from the gel (Fig. 2c). After chymotryptic digestion, the NT3 proteins were analyzed by LC-ETD-MS/MS, and the identified phosphorylation sites were compared between the shifted and non-shifted samples. Finally, we succeeded in identifying Ser575 as an ASK3-dependent phosphorylation site in the NT3 fragment (Fig. 2d). Ser575 was, to our knowledge, a novel phosphorylation site in WNK4. Unfortunately, the ASK3-dependent phosphorylation site in the CT1 fragment, which also displayed a mobility shift on SDS-PAGE, could not be specified (data not shown). Ser575 and its surrounding sequence are highly conserved between murine WNK4 and human WNK4 (Fig. 2e). Notably, Ser575 is the closest serine residue to the PHAII mutation hot spot in WNK4 (Fig. 2e).

To determine whether Ser575 is required for the ASK3-dependent mobility shift of the NT3 fragment, we examined the ASK3 overexpression-induced mobility shift of a series of NT3 fragment with Ser575 mutations. NT3-S575A (Ser->Ala mutant) did not exhibit the ASK3-dependent mobility shift. Moreover, the phospho-mimic mutants NT3-S575D (Ser->Asp mutant) and NT3-S575E (Ser->Glu mutant) exhibited a moderate, ASK3-independent mobility shift (Fig. 2f). These data strongly suggest that the phosphorylation of Ser575 is responsible for the ASK3-dependent mobility shift of the NT3 fragment.

### Ser575 is phosphorylated via the p38MAPK-MK pathway

**Figure 3:**
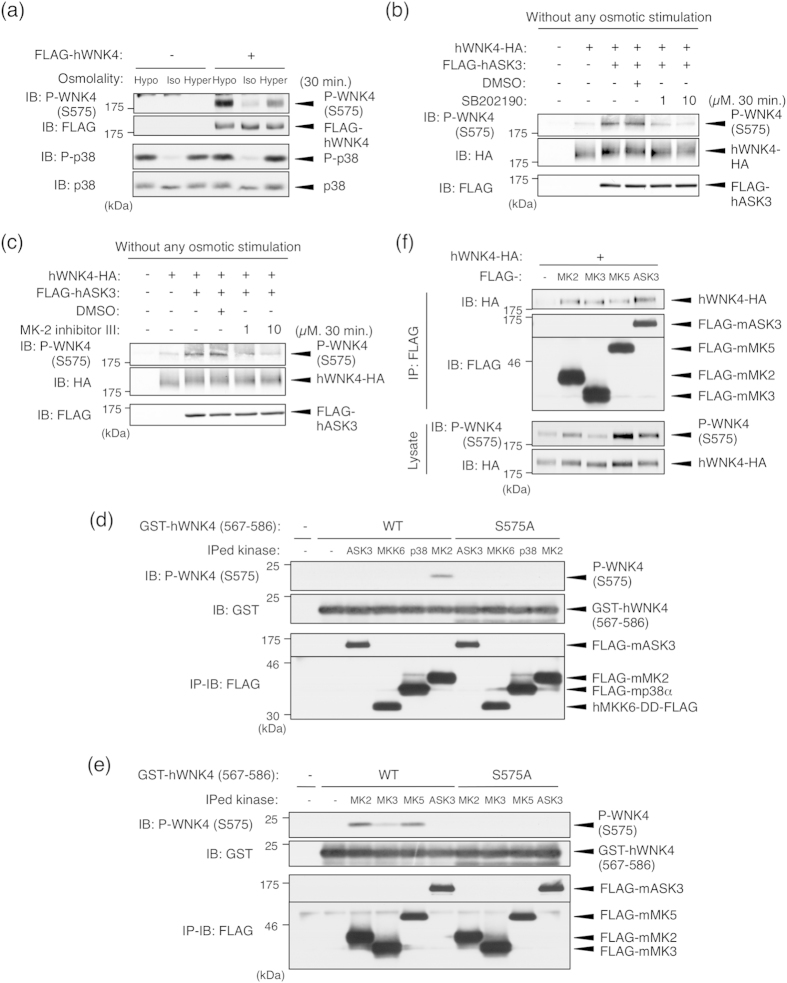
ASK3-dependent WNK4 Ser575 phosphorylation is mediated by the p38MAPK-MK pathway. (**a**) WNK4 Ser575 phosphorylation is induced by the osmotic stress. HEK293A cells were transfected with FLAG-tagged human WNK4 (FLAG-hWNK4). At 48 h after transfection, cells were stimulated with indicated stimulation buffer for 30 minutes. Full-length blots are presented in Supplementary Fig. S7a. (**b**) Ser575 phosphorylation induced by ASK3 co-expression without any osmotic stress is attenuated by p38MAPK inhibition. HEK293A cells were transiently transfected with hWNK4-HA and FLAG-hASK3. Before lysis, the cells were treated with DMSO or SB202190 at the indicated concentrations for 30 min. Full-length blots are presented in Supplementary Fig. S7b. (**c**) Ser575 phosphorylation induced by ASK3 co-expression without any osmotic stress is attenuated by MK2 inhibition. HEK293A cells were transiently transfected with hWNK4-HA and FLAG-hASK3. Before lysis, the cells were treated with DMSO or MK-2 inhibitor III at the indicated concentrations for 30 min. Full-length blots are presented in Supplementary Fig. S7b. (**d**) Only MK2 directly phosphorylates WNK4 Ser575 among the components of the ASK3-MKK6-p38MAPK-MK pathway. mIMCD3 cells were transiently transfected with FLAG-tagged mouse ASK3, a constitutively active human MKK6 mutant (MKK6-DD), mouse p38α and mouse MK2. Immunoprecipitated kinases were incubated with GST-hWNK4 (567–586) and Mg-ATP for 15 min at 30 °C. Full-length blots are presented in Supplementary Fig. S7c. (**e**) MK2, MK3 and MK5 are capable of directly phosphorylating WNK4 Ser575. mIMCD3 cells were transiently transfected with FLAG-tagged mouse MK2, mouse MK3 or mouse MK5. Immunoprecipitated kinases were incubated with GST-hWNK4 (567–586) and Mg-ATP for 15 min at 30 °C. Full-length blots are presented in Supplementary Fig. S7d. (**f**) Interaction between WNK4 and MK kinases. mIMCD3 cells were transiently transfected with hWNK4-HA and FLAG-tagged mouse MK2, MK3 and MK5. Cells were lysed at 24 h after transfection, and FLAG-tagged MK kinases were immunoprecipitated with anti-FLAG antibody beads. Full-length blots are presented in Supplementary Fig. S8.

In our previous study, we reported that ASK3 shows a bidirectional response to the osmotic stress: Hypotonic stimulation activates ASK3 whereas hypertonic stimulation inactivates it^23^. Several studies have revealed that the WNK-SPAK/OSR1 pathway also responds to the osmotic stress^24^,^25^,^26^. Therefore, we first examined whether WNK4 Ser575 is phosphorylated in an osmotic stress-dependent manner using a phospho-Ser575 specific antibody, which was raised against a phospho-peptide that encompasses the surrounding 12 amino acids of Ser575 on WNK4. The Ser575 phosphorylation of overexpressed WNK4 in HEK293A cells was markedly increased by both hypotonic and hypertonic stimulation (Fig. 3a). Since ASK3 is inactivated by hypertonic stimulation, this result suggested that WNK4 Ser575 is not a specific substrate of ASK3 and there should be another Ser575 kinase than ASK3 at least in the condition of hypertonic stimulation.

We noticed that the amino acid sequence surrounding Ser575 was identical to the substrate consensus sequence (ϕxRxx[pS]ϕ, where ϕ is a hydrophobic residue) of MAPK-activated protein kinase 2/3/5 (MK2/3/5), which are phosphorylated and activated by p38MAPK^27^,^28^. Moreover, p38MAPK was activated by both hypotonic and hypertonic stimulation (Fig. 3a). Given these findings, along with the fact that ASK3 is the upstream kinase of the p38MAPK pathway^23^, we investigated whether ASK3-dependent phosphorylation is mediated by the p38MAPK-MK pathway.

First, we examined the effect of a p38MAPK inhibitor (SB202190) and MK2 inhibitor (MK-2 inhibitor III) on the ASK3-dependent phosphorylation of WNK4 at Ser575 without any osmotic stress. ASK3 co-expression-dependent WNK4 Ser575 phosphorylation was attenuated by treatment with SB202190 (Fig. 3b), and was also attenuated by treatment with MK-2 inhibitor III in a dose-dependent manner (Fig. 3c).

Next, we performed an *in vitro* immune complex kinase assay to examine whether ASK3 and/or its downstream kinases were able to directly phosphorylate WNK4 at Ser575. We tested four different kinases, including ASK3 and kinases that are potentially activated downstream of ASK3 (MKK6, p38MAPK and MK2). In order to evaluate the kinase activity at the kidney epithelial cell where endogenous WNK4 is expressed, we used mIMCD3 cells that is an inner medullary collecting duct-derived cell line as a transfectant. ASK3 did not directly phosphorylate GST-hWNK4 (567–586), the recombinant substrate containing Ser575 residue; only MK2 phosphorylated Ser575 (Fig. 3d). Because this phospho-Ser575 signal was not detected when GST-hWNK4-S575A (567–586) was used as the substrate, the detected signal using our phospho-Ser575 specific antibody was confirmed to represent the phosphorylation of Ser575 of WNK4. Moreover, we examined whether other MK-family kinases (MK3 and MK5), whose substrate consensus sequences are the same as that of MK2, could phosphorylate Ser575^27^,^28^. WNK4 Ser575 phosphorylation was also detected *in vitro* when either MK3 or MK5 were used (Fig. 3e). This MK2-dependent phosphorylation of WNK4 Ser575 was also confirmed by the *in vitro* kinase assay in which a recombinant MK2 kinase was used (see Supplementary Fig. S3 online). Taken together, these results suggested that the ASK3-dependent phosphorylation of WNK4 at Ser575 was achieved via the p38MAPK-MK pathway.

In addition, we examined the interaction between WNK4 and MK2/3/5. Using a co-immunoprecipitation assay, we detected the interaction between WNK4 and MK2/3/5 in mIMCD3 cells (Fig. 3f). This result suggested that not only ASK3 but also the direct kinase MKs form a complex with WNK4.

### Osmotic stress induces WNK4 Ser575 phosphorylation via the p38MAPK-MK pathway and ASK3 is required for hypotonic stimulation-induced p38MAPK activation

**Figure 4:**
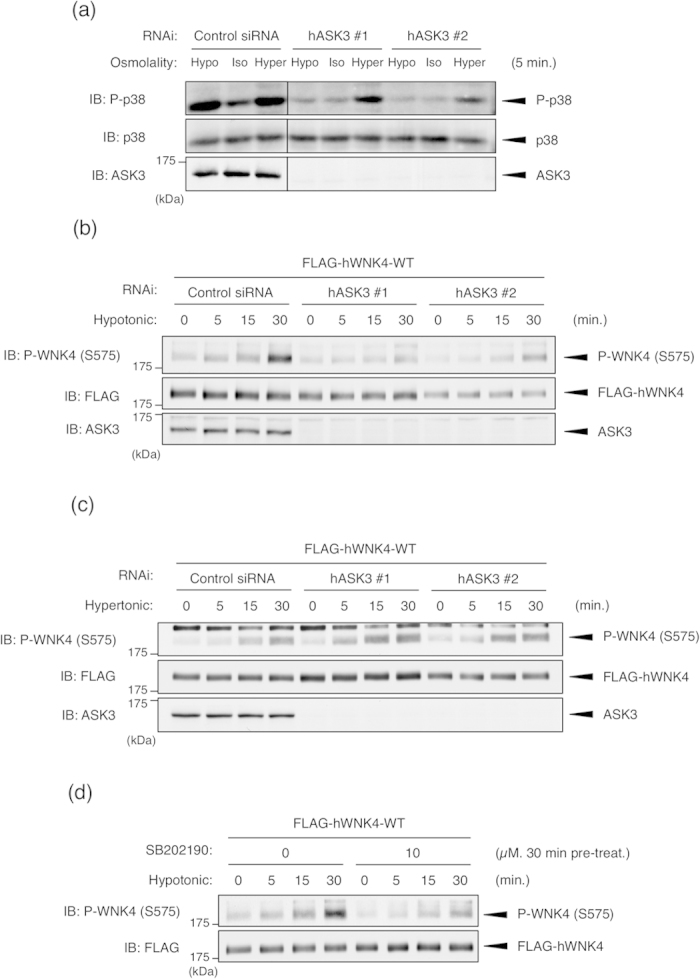
Osmotic stress induces WNK4 Ser575 phosphorylation via the p38MAPK-MK pathway and ASK3 is required for the hypotonic stimulation-dependent p38MAPK activation. (**a**) ASK3 is required for the hypotonic stimulation-dependent p38MAPK activation in HEK293A cells. HEK293A cells were transiently transfected with the indicated siRNA. At 48 h after transfection, cells were stimulated with indicated stimulation buffer for 5 minutes. Full-length blots are presented in Supplementary Fig. S9a. (**b**) ASK3 is required for the hypotonic stimulation-dependent WNK4 Ser575 phosphorylation. HEK293A cells were transiently transfected with FLAG-hWNK4-WT and the indicated siRNA. At 48 h after transfection, cells were stimulated with hypotonic buffer for the indicated times. Full-length blots are presented in Supplementary Fig. S9b. (**c**) ASK3 is not required for the hypertonic stimulation-dependent WNK4 Ser575 phosphorylation. HEK293A cells were transiently transfected with FLAG-hWNK4-WT and the indicated siRNA. At 48 h after transfection, cells were stimulated with hypertonic buffer for the indicated times. Full-length blots are presented in Supplementary Fig. S9c. (**d**) Hypotonic stimulation-dependent WNK4 Ser575 phosphorylation is attenuated by p38MAPK inhibition. HEK293A cells were transiently transfected with FLAG-hWNK4-WT. Before stimulation, cells were treated with DMSO or SB202190 at the indicated concentrations for 30 min. Cells were stimulated with hypotonic buffer for the indicated times. Full-length blots are presented in Supplementary Fig. S9d.

To address the involvement of ASK3 in the osmotic stress-dependent WNK4 Ser575 phosphorylation, we first examined whether ASK3 is involved in the osmotic stress-induced p38MAPK activation. ASK3 depletion by knockdown completely abolished the p38MAPK activation in hypotonic stimulation but not in hypertonic stimulation (Fig. 4a). This result is consistent with our previous data that ASK3 is activated by hypotonic stimulation and is inactivated by hypertonic stimulation^23^. The ASK3 knockdown experiment further revealed that ASK3 is required for the hypotonic stimulation-induced WNK4 Ser575 phosphorylation (Fig. 4b). On the other hand, ASK3 depletion did not affect the hypertonic stimulation-induced WNK4 Ser575 phosphorylation (Fig. 4c), which is consistent with the effect on p38MAPK (Fig. 4a). The hypotonic stimulation-induced WNK4 Ser575 phosphorylation is also inhibited by the treatment of SB202190 (Fig. 4d). Taken together, these results suggested that WNK4 Ser575 phosphorylation is induced by both hypertonic and hypotonic stress and endogenous ASK3 is involved in the phosphorylation of WNK4 Ser575 through p38MAPK activation only in the hypotonic stimulation.

### Hypotonic low-chloride stimulation induces WNK4 Ser575 phosphorylation via the p38MAPK-MK2 pathway

**Figure 5:**
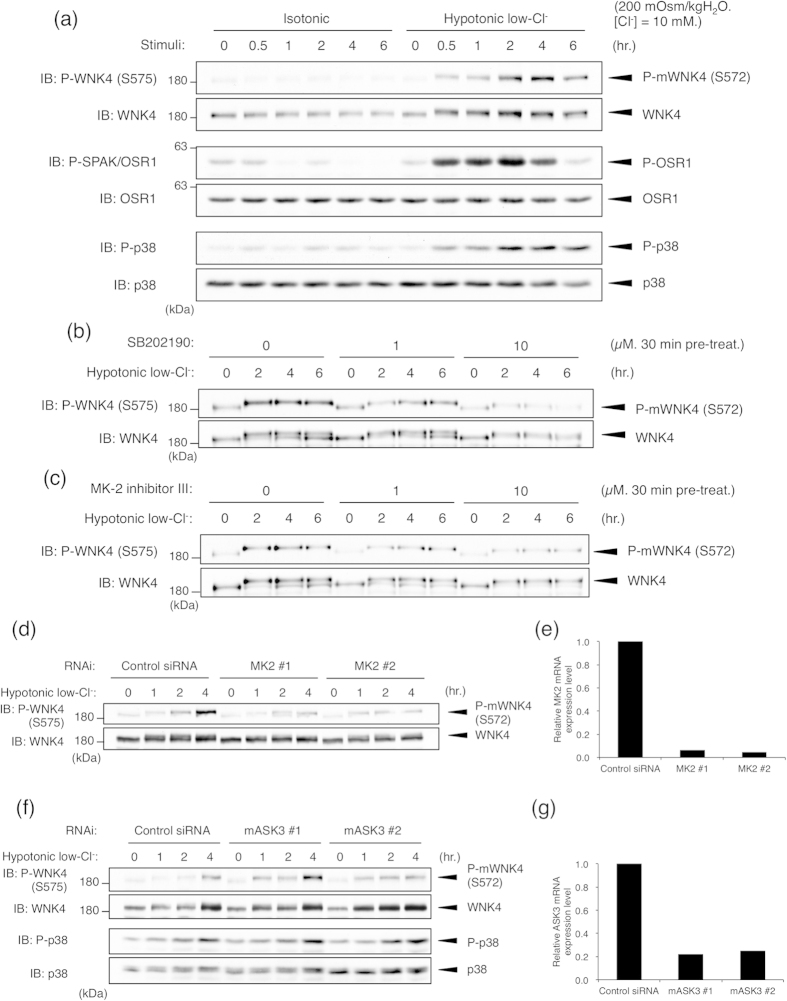
WNK4 in mpkDCT cell is phosphorylated at Ser572 in a hypotonic low-chloride stimulation-dependent manner via the p38MAPK-MK2 pathway. (**a**) WNK4 Ser572 (Ser575 in human WNK4) phosphorylation is induced by hypotonic low-chloride stimulation. mpkDCT cells were stimulated with hypotonic low-chloride buffer or isotonic buffer for the indicated times. Full-length blots are presented in Supplementary Fig. S10a. (**b**) Hypotonic low-chloride stimulation-induced WNK4 Ser572 phosphorylation is attenuated by p38MAPK inhibition. mpkDCT cells were pre-treated with DMSO or SB202190 at the indicated concentration for 30 min before stimulation. Cells were then stimulated with hypotonic low-chloride buffer for the indicated times. Full-length blots are presented in Supplementary Fig. S10b. (**c**) Hypotonic low-chloride stimulation-induced WNK4 Ser572 phosphorylation is attenuated by MK2 inhibition. mpkDCT cells were pre-treated with DMSO or MK-2 inhibitor III at the indicated concentration for 30 min before stimulation. Cells were then stimulated with hypotonic low-chloride buffer for the indicated times. Full-length blots are presented in Supplementary Fig. S10c. (**d**) Hypotonic low-chloride stimulation-induced WNK4 Ser572 phosphorylation is attenuated by MK2 knockdown. mpkDCT cells were transiently transfected with the indicated siRNA. At 96 h after transfection, cells were stimulated with hypotonic low-chloride buffer for the indicated times. Full-length blots are presented in Supplementary Fig. S11a. (**e**) Knockdown efficiency of MK2. mpkDCT cells were transiently transfected with the indicated siRNA. At 96 h after transfection, MK2 mRNA levels were analyzed by qRT-PCR. (**f**) Hypotonic low-chloride stimulation-induced p38MAPK activation and WNK4 Ser572 phosphorylation are not attenuated by ASK3 knockdown. mpkDCT cells were transiently transfected with the indicated siRNA. At 96 h after transfection, cells were stimulated with hypotonic low-chloride buffer for the indicated times. Full-length blots are presented in Supplementary Fig. S11b. (**g**) Knockdown efficiency of ASK3. mpkDCT cells were transiently transfected with the indicated siRNA. At 96 h after transfection, ASK3 mRNA levels were analyzed by qRT-PCR.

Hypotonic low-chloride stimulation is known to effectively activate WNK kinases, including WNK4^8^,^29^,^30^. The SPAK/OSR1-NCC pathway has also been reported to be activated by hypotonic low-chloride stimulation^29^,^31^,^32^. Thus, we next examined whether the phosphorylation status of WNK4 Ser575 might be altered by hypotonic low-chloride stimulation. When mpkDCT cells were stimulated with hypotonic low-chloride buffer, Ser572 phosphorylation (Ser575 in human WNK4) of endogenous WNK4 was clearly observed (Fig. 5a). Simultaneously, the activation of OSR1 and p38MAPK was also detected (Fig. 5a). The phosphorylation of Ser572 was effectively attenuated by treatment with either SB202190 or MK-2 inhibitor III (Fig. 5b,c). MK2 knockdown also attenuated the hypotonic low-chloride stimulation-dependent WNK4 Ser572 phosphorylation (Fig. 5d,e). Taken together, similar to HEK293A cells, these results suggested that endogenous WNK4 Ser572 is phosphorylated in a hypotonic low-chloride stimulation-dependent manner via the p38MAPK-MK2 pathway. However, unlike the result of the simple hypotonic stimulation in HEK293A cells, we did not observe an obvious requirement of ASK3 in the hypotonic low-chloride stimulation-dependent p38MAPK activation and WNK4 Ser572 phosphorylation (Fig. 5f,g). This result implied that the simple hypotonic stimulation and the hypotonic low-chloride stimulation are substantially different and that other MAP3Ks than ASK3 would be involved in the hypotonic low-chloride stimulation-dependent p38MAPK activation in mpkDCT cells.

### Ser575 phosphorylation is not involved in either hypotonic low-chloride stimulation-dependent WNK4 activation or the interaction with KLHL3

**Figure 6:**
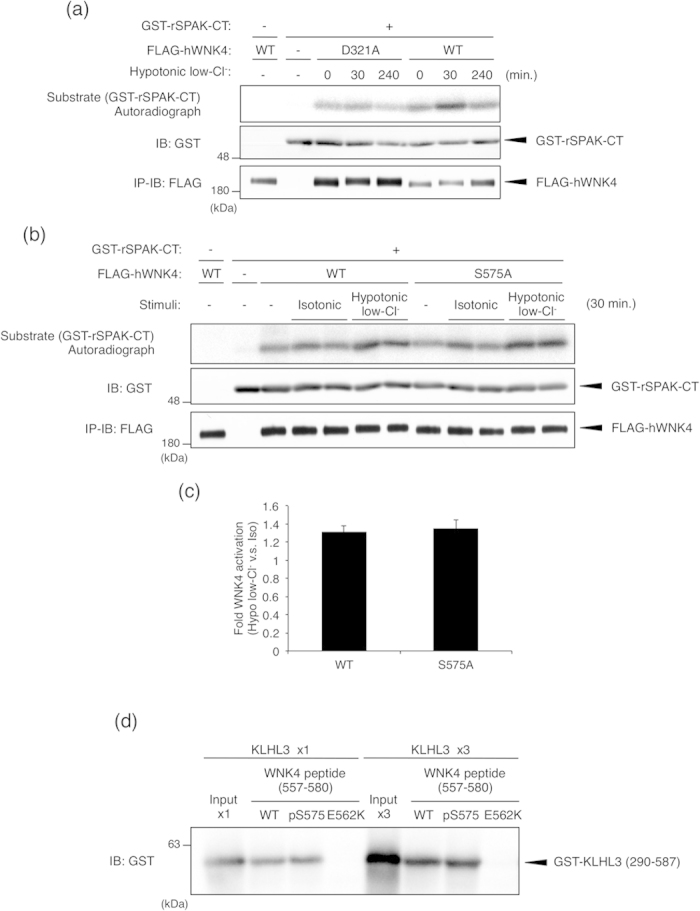
Ser575 phosphorylation is not critically involved in the regulation of WNK4 kinase activity and the binding affinity to KLHL3. (**a**) WNK4 kinase activity is increased in a hypotonic low-chloride stimulation-dependent manner. mpkDCT cells were infected with adenoviral vectors encoding FLAG-hWNK4-WT or the D321A mutant. At 48 h after infection, cells were lysed, and FLAG-tagged WNK4 proteins were immunoprecipitated with anti-FLAG antibody beads. Immunoprecipitated kinases were incubated with GST-rSPAK-CT (348–553) and Mg-ATP for 15 min at 30 °C. The reaction was terminated by the addition of SDS sample buffer, and samples were subjected to autoradiogram and IB. Full-length blots are presented in Supplementary Fig. S12a. (**b**) WNK4-WT and the WNK4-S575A mutant are equally activated by hypotonic low-chloride stimulation. mpkDCT cells were infected with adenoviral vectors encoding FLAG-hWNK4-WT or the S575A mutant. At 48 h after infection, cells were treated with isotonic or hypotonic low-Cl^−^ stimulation for 30 min. Then, cells were lysed, and FLAG-tagged WNK4 proteins were immunoprecipitated with anti-FLAG antibody beads. Immunoprecipitated kinases were incubated with GST-rSPAK-CT (348–553) and Mg-ATP for 15 min at 30 °C. The reaction was terminated by the addition of SDS sample buffer, and samples were subjected to autoradiogram and IB. Full-length blots are presented in Supplementary Fig. S12b. (**c**) Quantification of the hypotonic low-chloride stimulation-dependent WNK4 activation. The fold activation was calculated by dividing the intensity of autoradiogram in hypotonic low-Cl^−^ stimulation by that in isotonic stimulation in Fig. 5b. Error bars indicate SEM (N = 5). (**d**) *In vitro* interaction between KLHL3 and WNK4 (557–580) peptides. GST-KLHL3 (290–587) protein was pulled-down with beads that were coated with synthesized WNK4 (557–580) peptides. WT: the unphosphorylated peptide, pS575: the phosphorylated peptide at Ser575 and E562K: the peptide harboring PHAII mutation (a Glu to Lys substitution at 562 a.a.) as a negative control. The amount of pulled-down GST-KLHL3 was analyzed by IB. The input lane shows the total amount of GST-KLHL3 applied to the pull-down system. In the right four lanes, 3-fold higher amount of GST-KLHL3 was applied compared to that in the left four lanes. Full-length blots are presented in Supplementary Fig. S12c.

To examine the involvement of Ser575 phosphorylation in regulating WNK4 kinase activity, we established an *in vitro* kinase assay system for detecting the kinase activity of exogenously expressed WNK4 in mpkDCT cells. In this assay, we used GST-rSPAK-CT (348–553), which has been reported to contain a WNK-dependent phosphorylation site, as a substrate^7^,^8^. First, we examined whether WNK4 was activated in a hypotonic low-chloride stimulation-dependent manner. Using the established *in vitro* kinase assay system, we observed the clear activation of exogenously expressed WNK4-WT at 30 min after stimulation (Fig. 6a). This activation was not observed when WNK4-D321A, a kinase activity-deficient WNK4 mutant, was used as the kinase, excluding the concern that this assay system detected the activity of other contaminating kinases (Fig. 6a). Subsequently, we sought to determine whether the mutation of Ser575 affected the hypotonic low-chloride stimulation-dependent activation of WNK4. We observed no significant difference between WNK4-WT and WNK4–S575A in the fold-activation at 30 min after stimulation (Fig. 6b,c). These data suggest that Ser575 phosphorylation does not affect the hypotonic low-chloride stimulation-dependent activation of WNK4.

As described in Fig. 2e, Ser575 is the closest serine residue to the hot spot of PHAII-causing mutations. Recent studies found that the acidic region containing these mutation sites forms a binding site for KLHL3^15^, which is critical for regulating WNK4 protein levels via the ubiquitin-proteasome pathway^16^,^17^,^18^. Therefore, we next investigated whether Ser575 phosphorylation might affect the interaction of WNK4 with KLHL3 (Fig. 6d). To test this, recombinant GST-KLHL3 (290–587) was pulled down by synthetic WNK4 (557–580) peptides. GST-KLHL3 was efficiently pulled down by WNK4 (557–580)-WT peptide but not by WNK4 (557–580)-E562K, confirming that PHAII mutation disrupts the interaction with KLHL3^18^. Meanwhile, the binding affinity of WNK4 (557–580)-pS575 (peptide phosphorylated at Ser575) was almost identical to that of WNK4 (557–580)-WT (unphosphorylated peptide). We also detected no significant differences either in the interaction profile between WNK4 Ser575 full-length mutants and KLHL3 (see Supplementary Fig. S4a online) or in the KLHL3-dependent degradation profile of WNK4 mutants (see Supplementary Fig. S4b online). These results suggest that WNK4 Ser575 phosphorylation does not affect the interaction with KLHL3.

## Discussion

**Figure 7:**
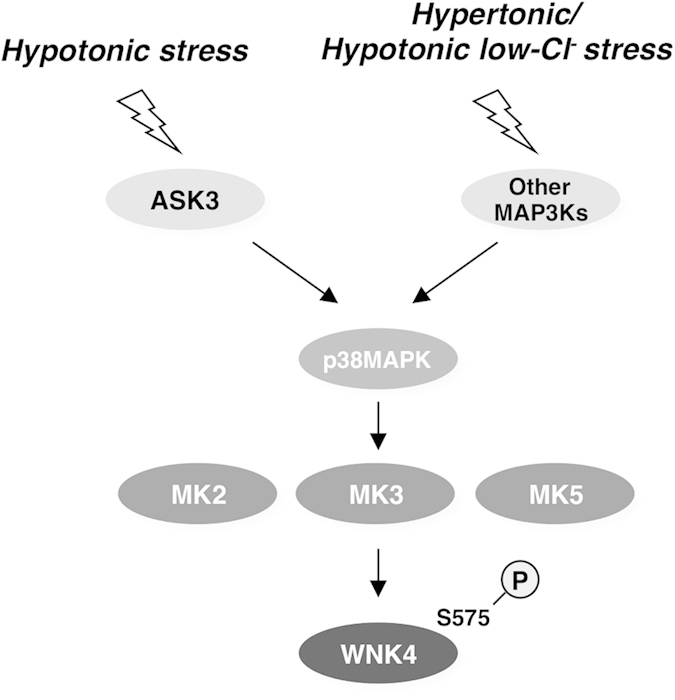
The schematic model of the identified signaling cascade toward the WNK4 Ser575 phosphorylation. When cells are exposed to osmotic stress, WNK4 Ser575 phosphorylation is increased via the p38MAPK-MK pathway. ASK3 is required for the p38MAPK activation induced by hypotonic stimulation. In case of hypertonic stimulation or hypotonic low-chloride stimulation, other MAP3K would be involved in triggering p38MAPK activation. However the function of Ser575 phosphorylation remains unknown, our results suggest that the p38MAPK-MK pathway might regulate WNK4 in an osmotic stress-dependent manner.

In this study, we identified Ser575 as a novel phosphorylation site in human WNK4. We revealed that the osmotic stress induces WNK4 Ser575 phosphorylation via the p38MAPK-MK pathway and ASK3 is required for the hypotonic stimulation-dependent p38MAPK activation, which leads to WNK4 Ser575 phosphorylation. Additionally, we revealed that, in mpkDCT cells, the phosphorylation of mouse WNK4 at Ser572 (Ser575 in human WNK4) is increased in a hypotonic low-chloride stimuli-dependent manner via the p38MAPK-MK pathway. Taken together, we propose a model in which the p38MAPK-MK pathway might be involved in the regulation of WNK4 in response to osmotic stress (Fig. 7). Although it has been reported that SPAK positively regulates p38MAPK^33^, our study provides the first evidence which suggests the role of the p38MAPK in the regulation of the WNK-SPAK/OSR1 pathway.

We succeeded in identifying Ser575 as a novel phosphorylation site in human WNK4. Murine WNK4 also contains conserved residues that correspond to Ser575 (Fig. 2e). However, other WNK kinases (WNK1, 2 and 3) do not possess serine residues that correspond to WNK4 Ser575, suggesting that Ser575 phosphorylation-dependent regulation of WNK4 is unique among WNK kinases.

The Ser575 residues of WNK4 PHAII mutants were phosphorylated similarly to WNK4-WT upon ASK3 overexpression (data not shown). Nevertheless, we cannot exclude the possibility that the PHAII mutation affects the Ser575 phosphorylation-mediated regulation of WNK4 without affecting Ser575 phosphorylation itself.

Given that WNK4 was identified as an ASK3-interacting protein in a yeast two-hybrid screen, which usually represents direct protein-protein interactions, we speculated that ASK3 directly phosphorylated WNK4 Ser575. However, *in vitro* immune complex kinase assays revealed that WNK4 Ser575 was not phosphorylated directly by ASK3 (Fig. 3d). This suggests that the interaction between ASK3 and WNK4 was not indicative of an enzyme-substrate interaction. One possible interpretation of this interaction is that ASK3 itself serves as a scaffold for the ASK3-MKK3/6-p38MAPK-MK-WNK4 pathway. Considering that high salt diet-treated ASK3 knockout mice exhibit the SPAK/OSR1-NCC pathway hyperactivation in the kidney^23^, it is possible that ASK3 functions as an upstream kinase of the p38MAPK-MK-WNK4 Ser575 pathway in the kidney. This point is discussed further below.

We also showed that ASK3 co-expression-, osmotic stress- and the hypotonic low-chloride stimulation-dependent WNK4 Ser575 phosphorylation are mediated via the p38MAPK-MK pathway. In mammals, there are three closely related MK kinases (MK2, MK3 and MK5)^27^,^28^. We showed that all three of these kinases could directly phosphorylate Ser575 *in vitro* (Fig. 3e). Because MK-2 inhibitor III, which we used in this study to inhibit MK2, probably exerts some effect to MK3 and MK5 (IC_50_ of 8.5 nM, 210 nM and 81 nM against MK2, MK3 and MK5, respectively)^34^, the data using MK-2 inhibitor III in this study can be interpreted to represent the inhibition of all three MK kinases. Considering the results of the MK2 knockdown experiment, MK2 seems to play major role in hypotonic low-chloride stimulation-dependent Ser575 phosphorylation (Fig. 5d,e).

What is the physiological function of Ser575 phosphorylation? One possibility was the regulation of WNK4 kinase activity. We examined the involvement of Ser575 phosphorylation in regulating WNK4 kinase activity using an *in vitro* immune complex kinase assay, only to find that there was no significant difference in hypotonic low-chloride stimulation-dependent kinase activation between WNK4-WT and WNK4-S575A (Fig. 6b,c). These data suggest that Ser575 phosphorylation is not critically involved in the regulation of WNK4 kinase activity.

The WNK4 amino acid sequence contains a coiled-coil domain near the Ser575 residue, which is often involved in protein-protein interactions. Based on this information, it is also possible that Ser575 phosphorylation affects protein-protein interactions mediated by the coiled-coil domain. Recently, it was reported that the binding of WNK4 to KLHL3, a substrate-recognition protein of the Cullin3-based E3 ligase complex, is disrupted by PHAII mutations, leading to increase of WNK4 protein levels^18^. Because the Ser575 residue lies close to the binding region for KLHL3^15^, we investigated whether Ser575 phosphorylation might alter the interaction with KLHL3 using pull-down assays (Fig. 6d). However, both unphosphorylated and phosphorylated WNK4 peptides interacted with KLHL3 to a similar degree, suggesting that Ser575 phosphorylation does not critically affect the WNK4-KLHL3 interaction. It would be interesting to explore which WNK4-interacting molecule is affected by the Ser575 phosphorylation.

The Ser575 residue is also close to a region enriched with negatively charged amino acids. Like an EF hand motif, the negatively charged domain potentially senses positively charged calcium ions^35^,^36^. A recent study reported that PHAII mutations disrupt calcium ion-dependent WNK4 regulation^37^. Moreover, WNK4 PHAII mutants have been shown to lose sensitivity to angiotensin II stimulation, which triggers calcium signaling^38^,^39^. These data suggest the calcium ion-dependent regulation of WNK4 through the negatively charged domain, and this effect could also potentially involve the Ser575 phosphorylation site.

In this study, ASK3 overexpression was revealed to induce WNK4 phosphorylation at Ser575 via the p38MAPK-MK pathway (Fig. 3). The knockdown experiment showed that ASK3 is required for the hypotonic stimulation-dependent p38MAPK activation and WNK4 Ser575 phosphorylation (Fig. 4a,b). This indicates that ASK3 can function as an upstream MAP3K that phosphorylates WNK4 Ser575 by activating the p38MAPK-MK pathway at least in the hypotonic stress condition. In case of the hypertonic stimulation, it is reported that the Rac-MEKK3-MKK3 signaling pathway is involved in the p38MAPK activation^40^. From this report, it is possible to discuss that MEKK3 might be an upstream MAP3K that induces the hypertonic stimulation-dependent WNK4 Ser575 phosphorylation.

In our previous study, we have shown that ASK3 inhibits the hypotonic stimulation-induced OSR1 activation in HeLa cells via the inhibition of WNK1 activity^23^. We consider that the p38MAPK-MK pathway-dependent WNK4 phosphorylation revealed in this study is independent of the negative regulation of the WNK1-OSR1 pathway by ASK3 that we reported in our previous study for two reasons: First, other WNK kinases than WNK4 do not possess serine residues that correspond to WNK4 Ser575. Second, the inhibitory effect of ASK3 on the WNK1-OSR1 pathway observed in HeLa cells was not abolished by the treatment of SB202190^23^.

ASK3 protein is highly expressed in the kidney^23^. High-salt diet challenge has been shown to induce p38MAPK activation in the rat kidney^41^. Moreover, we have reported that ASK3 knockout mice show hypertensive phenotypes when they are given a high-salt diet^23^. Therefore, it is possible that ASK3 is activated by high-salt diet challenge and subsequently activates the p38MAPK-MK-WNK4 Ser575 signaling pathway in the kidney. It would be interesting to investigate whether the activation of the p38MAPK pathway in the kidney via high-salt diet challenge is impaired in ASK3 knockout mice.

In conclusion, we provide the first evidence that WNK4 is phosphorylated downstream of the p38MAPK-MK pathway. This finding proves insights into a novel stress-dependent mechanism of WNK4 regulation.

## Methods

### Cell culture and transfection

HEK293A cells were cultured in Dulbecco’s modified Eagle’s medium with 4.5 mg/mL glucose containing 10% fetal bovine serum and 100 units/mL penicillin G. mIMCD3 cells were cultured in Dulbecco’s modified Eagle’s medium/nutrient mixture F-12 Ham containing 10% fetal bovine serum. mpkDCT cells were cultured in Dulbecco’s modified Eagle’s medium/nutrient mixture F-12 Ham containing 2% fetal bovine serum, 5 μg/mL Insulin, 50 nM Dexamethasone, 60 nM Selenium, 5 μg/mL Transferrin, 1 nM Triiodothyronine, 10 ng/mL EGF, 20 mM HEPES, 2 mM Glutamine, 2 mg/mL glucose. All cells were kept in a 5% CO_2_ atmosphere at 37 °C.

Transfection of plasmids into HEK293A cells was performed using FuGENE6 (Promega) or Polyethyleneimine-MAX (Polysciences). Transfection of plasmids into mIMCD3 cells was performed using Lipofectamine2000 (Life technologies).

For RNAi experiments, cells were transfected with the following siRNAs (Life technologies) using Lipofectamine RNAiMAX (Life technologies) according to the manufacturer’s instructions: human ASK3 #1 (CACCGAAGAGCAGUGCAGUAGAUUU), human ASK3 #2 (CGGAUUUCAGGAUGCCGUAAAUAAA), mouse ASK3 #1 (CACCGAAGACCAGUGUAAUAGAUUU), mouse ASK3 #2 (CAAGUACGGAUAGCCAUCAAGGAAA), mouse WNK4 #1 (AGAGUGCGUUGUGUUGUAUCCUGAA), mouse WNK4 #2 (CAGCUACUCAUCAACUACAUCUGAU), mouse MK2 #1 (UAGUACGGUGUAUAACAAGGAGUGG), mouse MK2 #2 (UCCUGGAUUCGGCUAAAGAGUUCUC), Stealth RNAi Negative CTL Medium GC Duplex #2 (Life technologies).

### Plasmids

The human WNK4-WT construct was kindly gifted by Dr. Shinichi Uchida (Tokyo Medical and Dental Univ.). For constructing FLAG-hWNK4-NT1 (1–166)/-NT2 (167–443)/-NT3 (444–645)/-CT1 (646–952)/-CT2 (953–1243), the following oligonucleotides were used. The nucleotides shown in the lowercase include sequences for digestion with restriction enzymes: NT1, sense, 5′-gcgaattcATGTTGGCATCCCCGGCCACGG-3′, antisense, 5′-gcctcgagTGCCACAGCCTGGGTCTCCATG-3′; NT2, sense, gcgatatcggACGTCCCCCGATGGCCGATA-3′, antisense, 5′-gcctcgagCGCTAGTTCCACGTGCACACC-3′; NT3, sense, 5′-gcgaattcGAGGAGGACGACGGCGAGAAGC-3′, antisense, 5′-gcctcgagATCTGAGGCATAGCTGTCCCCG-3′; CT1, sense, 5′-gcgaattcGCAGCTTCAGGCCTTAGCGATG-3′, antisense, 5′-gcctcgagACTGGGAAAGGAGCCCAGGTG-3′; CT2, sense, 5′-gcgaattcTCTCCTCCAGCCCCTCCTAGTC-3′, antisense, 5′-gcctcgagTCACATCCTGCCAACATCCCCG-3′. The mouse MK2 cDNA was amplified from a C57BL/6 mouse kidney cDNA library. The isolated mMK2 cDNA was re-amplified with the primers containing digestion sites. The mouse MK3 and MK5 cDNAs were amplified from an mpkDCT cDNA library. The following oligonucleotides were used: mouse MK2 (1^st^ primers), sense, 5′- ATGCTGTCGGGCTCTCCG-3′, antisense, 5′- TCAGTGGGCGAGAGCCG-3′; mouse MK2 (2^nd^ primers), sense, 5′- gggatatccaATGCTGTCGGGCTCTCCG-3′, antisense, 5′- ggctcgagTCAGTGGGCGAGAGCCG-3′; mouse MK3, sense, 5′- gggaattcATGGATGGCGAGACAGCAGG-3′, antisense, 5′- ggctcgagTTACTGGTTGTTGCATCCTTGTG-3′; mouse MK5, sense, 5′- gggaattcATGTCGGAGGACAGCGAC-3′, antisense, 5′- ggctcgagCTACTGGGGCTCGTGGG-3′. FLAG-hWNK4-NT3-S575A/S575D/S575E mutants were constructed according to the protocol for the *Dpn*I-mediated site-directed mutagenesis: S575A, sense, 5′- CCTTTTCCGCCACGCCGCCTACTCATCTACC-3′, antisense, 5′-GGTAGATGAGTAGGCGGCGTGGCGGAAAAGG-3′; S575D, sense, 5′- CCTTTTCCGCCACGCCGACTACTCATCTACC-3′, antisense, 5′- GGTAGATGAGTAGTCGGCGTGGCGGAAAAGG-3′; S575E, sense, 5′- CCTTTTCCGCCACGCCGAGTACTCATCTACC-3′, antisense, 5′- GGTAGATGAGTACTCGGCGTGGCGGAAAAGG-3′. For constructing GST-hWNK4 (567–586)-WT/S575A, hWNK4 (567–586)-WT/S575A sequences were amplified by the following oligonucleotides: sense, 5′-gggaattcCAGCCCTTCCTTTTCCGC-3′, antisense, 5′-ggctcgagTCAGTCTCGCAATCCGAAG-3′. For constructing GST-hKLHL3 (290-587), hKLHL3 (290–587) sequence was amplified using plasmid containing hKLHL3 cDNA (kindly gifted by Dr. Shinichi Uchida) by using following oligonucleotides: sense, 5′-gcgaattcAGGACCAAGCCCAGGACTCC-3′, antisense, 5′-gcctcgagTCACAAGGACTTGTGAATCAC-3′. Recombinant adenoviruses encoding FLAG-hWNK4-WT/S575A constructs were generated using Gateway technology (Invitrogen) according to the manufacturer’s instruction. FLAG-hASK3-WT, FLAG-hASK3-K681M, FLAG-mASK3-WT, hMKK6-S207D/T221D-FLAG, FLAG-mp38α and GST-rSPAK-CT (348–553) were described previously^23^,^42^,^43^.

### Quantitative reverse transcription (RT)-PCR

Total RNA was isolated from siRNA-treated mpkDCT cells using Isogen (Wako) and reverse-transcribed with the ReverTra Ace qPCR RT Master Mix with gDNA Remover (TOYOBO). Quantitative PCR was performed with Power SYBR Green PCR Master Mix (Roche) on an ABI PRISM 7000 Sequence Detection System (Applied Biosystems). The following oligonucleotides were used for amplifying *MAP3K15, MAPKAPK2* and *S18* sequences: *MAP3K15*, left, 5′-GAAATCCCAGAGAGAGATATCAGG-3′, right, 5′-TGTTTGAGATACTTGTGCAGAGC-3′; *MAPKAPK2*, left, 5′-CAGCAAAAATTCGCCCTAAA-3′, right, 5′-AGTGCAGCTCCACCTCTCTG-3′; *S18*, left, 5′-TCCAGCACATTTTGCGAGTA-3′, right, 5′-CAGTGATGGCGAAGGCTATT-3′.

### Generation of recombinant GST proteins

*Escherichia coli* strain BL21 cells were transformed by plasmids encoding GST-hWNK4 (567–586)-WT/S575A, GST-rSPAK-CT (348–553) or GST-hKLHL3 (290–587). Protein expression was induced by incubating with 0.1 mM Isopropyl-1-thio-β-galactopyranoside (IPTG) for 2 hours at 30 °C. Purified proteins on a glutathione-Sepharose column were eluted with elution buffer (20 mM glutathione, 50 mM Tris-HCl pH 8.0).

### Antibodies

Rabbit polyclonal antibody to WNK4 (amino acids in mouse WNK4 44–58; RRFSGKAEPRPRSSR) and phospho-WNK4 Ser575 (amino acids in human WNK4 572–583; RHA[pS]YSSTTSDC) were raised as described previously^44^. The polyclonal antibody to phospho-ASK1 Thr845 was described previously^44^. The monoclonal antibodies to mouse ASK3, the monoclonal antibody to human ASK3 and the polyclonal antibody to phospho-SPAK/OSR1 were described previously^23^. Monoclonal antibody to FLAG-tag (1E6) was purchased from Wako Pure Chemical Industries. Monoclonal antibody to HA-tag (3F10) was purchased from Roche Applied Science. Phospho-specific antibody to p38MAPK (P-p38; Thr180/Thr182) was purchased from Cell Signaling. Polyclonal antibody to p38MAPK (C-20-G) was purchased from Santa Cruz. The antibodies to OSR1 (M06 and M01) were purchased from Abnova. Secondary antibodies against rabbit IgG and rat IgG were purchased from Cell Signaling. Secondary antibody against mouse IgG was purchased from GE Healthcare. Secondary antibody against goat IgG was purchased from Vector.

### Stimulation buffers

Isotonic buffer was 130 mM NaCl, 2 mM KCl, 1 mM KH_2_PO_4_, 2 mM CaCl_2_, 2 mM MgCl_2_, 10 mM glucose, 20 mM mannitol, 10 mM HEPES pH 7.4 with NaOH. Hypotonic buffer was same as isotonic buffer except for 90 mM NaCl and 0 mM mannitol. Hypertonic buffer was same as isotonic buffer except for 220 mM mannitol. Hypotonic low chloride buffer was same as hypotonic buffer except for 90 mM Na-gluconate.

### Yeast two-hybrid screening

A human kidney cDNA library (pre-transformed human kidney MATCHMAKER cDNA library; Clontech) was screened for proteins that interact with human ASK3-K681 M. The bait plasmid (pGBKT7; Clontech) expressing ASK3 protein was constructed in-frame with the GAL4 DNA-binding domain and transformed into MATa yeast strain AH109. Prey plasmids (pACT2; Clontech) including cDNA library fused with GAL4 activation domain were pre-transformed in MATα yeast strain Y187 (Clontech). These two strains formed diploid cells by mating and resulting clones were selected by selection medium (depleted of adenine, His, Leu and Trp). Plasmids of positive clones were recovered, and the cDNA inserts were sequenced.

### Identification of phosphorylation site by LC-ETD-MS/MS analysis

HEK293A cells were transiently transfected with FLAG-tagged human WNK4-NT3 (444-645 in a.a.) in combination with human ASK3-WT or ASK3-K681M mutant. At 48 hours after transfection, cells were lysed with IP lysis buffer (20 mM Tris-HCl pH 7.5, 150 mM NaCl, 10 mM EDTA pH 8.0, 1% sodium deoxycholate, 1% Triton X-100, 1 mM phenylmethylsulfonyl fluoride, 5 μg/mL leupeptin, 8 mM NaF, 12 mM β-glycerophosphate, 1 mM sodium orthovanadate, 1.2 mM sodium molybdate, 5 μM cantharidin, 2 mM imidazole). The lysates were immunoprecipitated with anti-FLAG antibody beads (M2 beads; Sigma) followed by washing two times with wash buffer 1 (20 mM Tris-HCl pH 7.5, 500 mM NaCl, 5 mM EGTA pH 7.5, 1% Triton X-100) and two times with wash buffer 2 (20 mM Tris-HCl pH 7.5, 150 mM NaCl, 5 mM EGTA pH 7.5). The immune complex was incubated with 25 mM iodoacetamide for 20 minutes before subjecting to SDS-PAGE. The bands of WNK4-NT3s were excised from the gel and digested with chymotrypsin for 16 hours at 37 °C. The digested peptides were subjected to LC-ETD-MS/MS analysis using Orbitrap Elite (Thermo Fisher Scientific).

### Cell stimulation, immunoblotting

Cells were stimulated with indicated stimulation buffers for indicated times. The cells were lysed in the IP lysis buffer or RIPA buffer (50 mM Tris-HCl pH 8.0, 150 mM NaCl, 1% NP-40, 0.5% sodium deoxycholate, 0.1% SDS, 1 mM phenylmethylsulfonyl fluoride, 5 μg/mL leupeptin, 8 mM NaF, 12 mM β-glycerophosphate, 1 mM sodium orthovanadate, 1.2 mM sodium molybdate, 5 μM cantharidin, 2 mM imidazole). The lysates were dissolved with 2× SDS sample buffer (100 mM Tris-HCl pH 8.8, 10% bromophenol blue, 36% glycerol, 4% SDS, 10 mM dithiothreitol). Alternatively, cells were lysed directly with 2× SDS sample buffer and were homogenized with the ultrasonic homogenizer. After boiling at 98°C for 3 minutes, the samples were subjected to SDS-PAGE and transferred to PVDF membranes. After blocking with 1% non-fat dried skimmed milk powder in TBS-T (137 mM NaCl, 50 mM Tris-HCl pH 8.0, 0.05% Tween 20), the membranes were probed with appropriate primary antibodies and secondary antibodies. The signals were detected by chemiluminescence reaction using luminol.

### *In vitro* immune complex kinase assay

Cells were lysed with IVK lysis buffer (20 mM Tris-HCl pH 7.5, 150 mM NaCl, 5 mM EGTA pH 7.5, 1% sodium deoxycholate, 1% Triton X-100, 12 mM β-glycerophosphate, 1 mM phenylmethylsulfonyl fluoride, 5 μg/mL leupeptin, 1 mM dithiothreitol, PhosSTOP (Roche)). The lysates were immunoprecipitated with anti-FLAG antibody beads followed by washing two times with wash buffer 1, one time with wash buffer 2 and one time with IVK kinase buffer (20 mM Tris-HCl pH 7.5, 20 mM MgCl_2_). The immune complexes were incubated in IVK kinase buffer containing 100 μM ATP together with 0.5 μg GST-hWNK4 (567–586) or GST-rSPAK-CT (348–553) at 30 °C for 15 minutes. Kinase reactions were terminated by adding 50 μL 2× Sample buffer followed by boiling at 98 °C for 3 minutes.

### Pull-down assay

FLAG-tagged WNK4 (557–580) peptides which contain the binding region to KLHL3^15^ were synthesized and purchased from Eurofins Genomics Inc. We designed three kinds of WNK4 (557–580) peptides: (I) unphosphorylated WT sequence, WNK4 (557–580)-WT; (II) WT sequence with phosphorylation at Ser575, WNK4 (557–580)-pS575; (III) E562K mutation (PHAII mutation) in the WT sequence, WNK4 (557–580)-E562K. The peptides were dissolved in PBS at 0.1 mg/ml. Each peptide (1 μg) was incubated with anti-FLAG antibody beads for 30 min followed by washing with IP lysis buffer. Subsequently, GST-hKLHL3 (290–587) was incubated with the WNK4 (557–580) peptide-coated beads for 6 hours followed by washing with IP lysis buffer. The GST-hKLHL3 protein bound to WNK4 peptides was dissolved with 2× Sample buffer containing 20 mM DTT.

## Additional Information

**How to cite this article**: Maruyama, J. *et al.* Osmotic stress induces the phosphorylation of WNK4 Ser575 via the p38MAPK-MK pathway. *Sci. Rep.* **6**, 18710; doi: 10.1038/srep18710 (2016).

## Supplementary Material

Supplementary Information
