# Supplementary material for: Osmotic stress induces the phosphorylation of WNK4 Ser575 via the p38MAPK-MK pathway
Source: Sci Rep. 2016 Jan 6;6:18710. doi: 10.1038/srep18710 (PMC4702109; doi:10.1038/srep18710)

Osmotic stress induces the phosphorylation of WNK4 Ser575 via the p38MAPK-MK pathway

Junichi Maruyama[1, 2], Yumie Kobayashi[1], Tsuyoshi Umeda[1], Alain Vandewalle[3], Kohsuke Takeda[1, 4], Hidenori Ichijo[*1] and Isao Naguro[*1]

[1]Laboratory of Cell Signaling, Graduate School of Pharmaceutical Sciences, The University of Tokyo, 7-3-1 Hongo, Bunkyo-ku, Tokyo 113-0033, Japan

[2]Department of Medical Biochemistry, Graduate School of Medical and Dental Sciences, Tokyo Medical and Dental University, 1-5-45 Yushima, Bunkyo-ku, Tokyo 113-8510, Japan

[3]Centre de Recherche sur l'Inflammation, UMRS 1149, Université Paris 7, site Bichat, 75018, Paris, France

[4]Cell Regulation, Graduate School of Biomedical Sciences, Nagasaki University, 1-14 Bunkyo-machi, Nagasaki 852-8521, Japan

[*]To whom correspondence should be addressed:

Isao Naguro. Tel: +81-3-5841-4858; Fax: +81-3-5841-4798; E-mail: nagurois@mol.f.u-tokyo.ac.jp

Hidenori Ichijo. Tel: +81-3-5841-4858; Fax: +81-3-5841-4798; E-mail: ichijo@mol.f.u-tokyo.ac.jp

Legends for the Supplementary Figures

Supplementary Figure S1. Osmotic stress-dependency of the ASK3-WNK4 interaction

Osmotic stress-dependency of the ASK3-WNK4 interaction. HEK293A cells were transiently transfected with FLAG-hASK3 and hWNK4-HA. Cells were lysed at 48 h after transfection. Before the lysis, cells were stimulated with the osmotic stimulation buffer as indicated for the indicated times. Lysates were immunoprecipitated with anti-FLAG antibody beads and subjected to IB.

Supplementary Figure S2. Profile of the interaction between WNK4 fragments and ASK3

HEK293A cells were transiently transfected with HA-hASK3 and FLAG-hWNK4 fragments. Cells were lysed at 48 h after transfection. Lysates were immunoprecipitated with anti-FLAG antibody beads and subjected to IB.

Supplementary Figure S3. Recombinant MK2 phosphorylates WNK4 Ser575 *in vitro*

0.5 μg recombinant MK2 (R&D systems) were incubated with 0.5 μg GST-hWNK4 (567-586) and Mg-ATP for 15 min at 30ºC. GST was used as a negative control for recombinant MK2 kinase. The reaction was terminated by the addition of SDS sample buffer, and samples were subjected to IB. Phosphorylation of Ser575 residue in GST-hWNK4 (567-586) was analyzed by IB using phospho-Ser575 antibody. Protein amount of recombinant MK2, GST and GST-hWNK4 (567-586) were visualized by Ponceau S staining.

Supplementary Figure S4. Involvement of WNK4 Ser575 phosphorylation in the KLHL3-dependent WNK4 regulation

(a) Interaction between WNK4 mutants and KLHL3. HEK293A cells were transiently transfected with FLAG-hWNK4 mutants and HA-KLHL3. Cells were lysed at 48 h after transfection. Lysates were immunoprecipitated with anti-FLAG antibody beads and subjected to IB.

(b) KLHL3-dependent degradation of WNK4 mutants. HEK293A cells were transiently transfected with FLAG-hWNK4 mutants and HA-KLHL3. Cells were lysed at 48 h after transfection. Before lysis, cells were treated with 5 μg/ml cycloheximide for the indicated times to inhibit the protein synthesis. Cell lysates were subjected to IB.

Supplementary Figure S5. Full-length blots of Figure 1

(a) Full-length blots of Figure 1b.

(b) Full-length blots of Figure 1c.

Supplementary Figure S6. Full-length blots of Figure 2

(a) Full-length blots of Figure 2b.

(b) Full-length blots of Figure 2f.

Supplementary Figure S7. Full-length blots of Figure 3a-e

(a) Full-length blots of Figure 3a.

(b) Full-length blots of Figure 3b-c.

(c) Full-length blots of Figure 3d.

(d) Full-length blots of Figure 3e.

Supplementary Figure S8. Full-length blots of Figure 3f

Full-length blots of Figure 3f.

Supplementary Figure S9. Full-length blots of Figure 4

(a) Full-length blots of Figure 4a.

(b) Full-length blots of Figure 4b.

(c) Full-length blots of Figure 4c.

(d) Full-length blots of Figure 4d.

Supplementary Figure S10. Full-length blots of Figure 5a-c

(a) Full-length blots of Figure 5a.

(b) Full-length blots of Figure 5b.

(c) Full-length blots of Figure 5c.

Supplementary Figure S11. Full-length blots of Figure 5d,f

(a) Full-length blots of Figure 5d.

(b) Full-length blots of Figure 5f.

Supplementary Figure S12. Full-length blots of Figure 6

(a) Full-length blots of Figure 6a.

(b) Full-length blots of Figure 6b.

(c) Full-length blots of Figure 6d.

Supplementary Figure S1

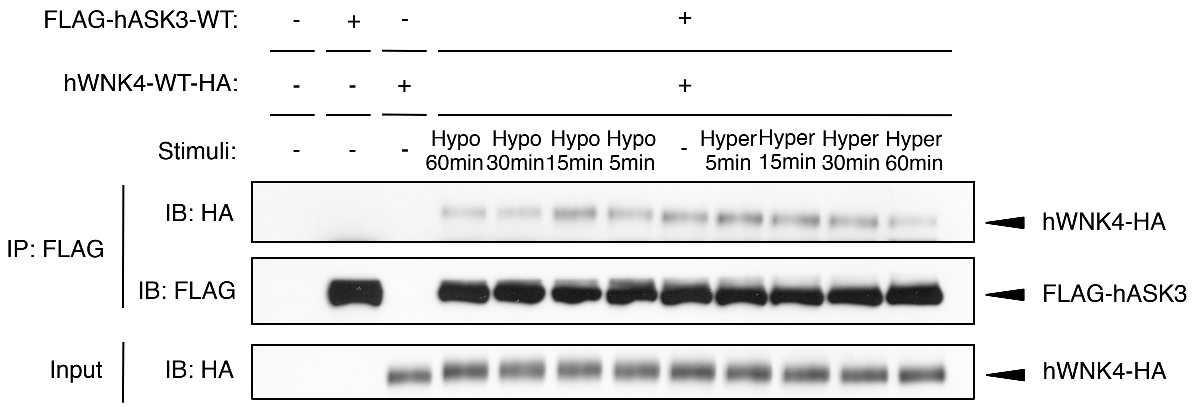

Supplementary Figure S2

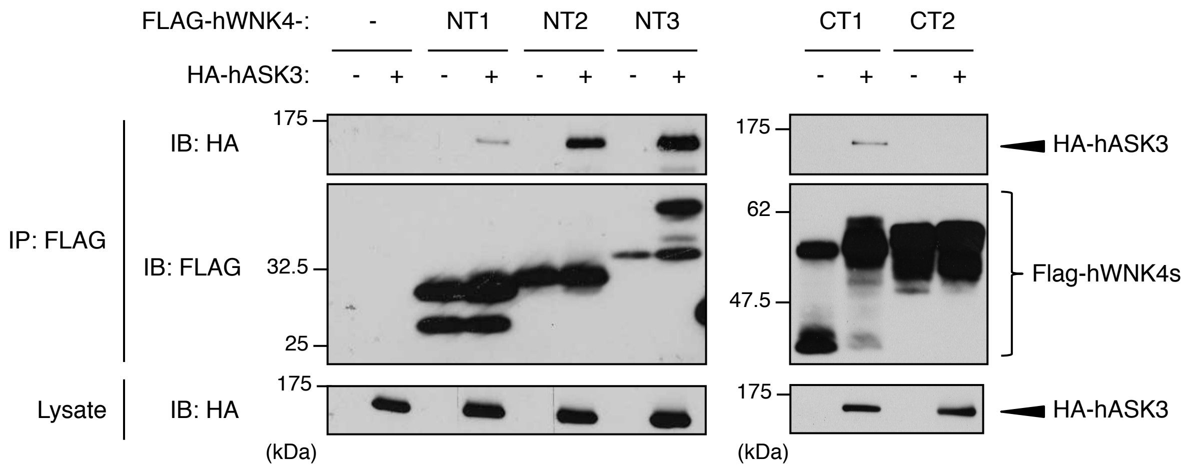

Supplementary Figure S3

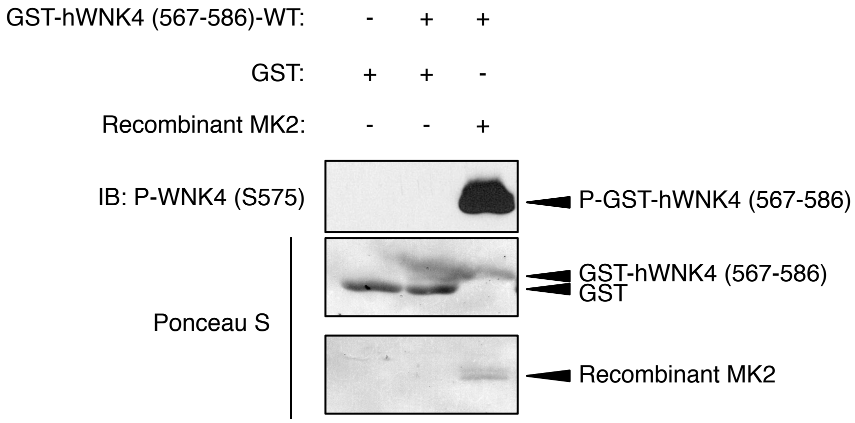

Supplementary Figure S4

(a)

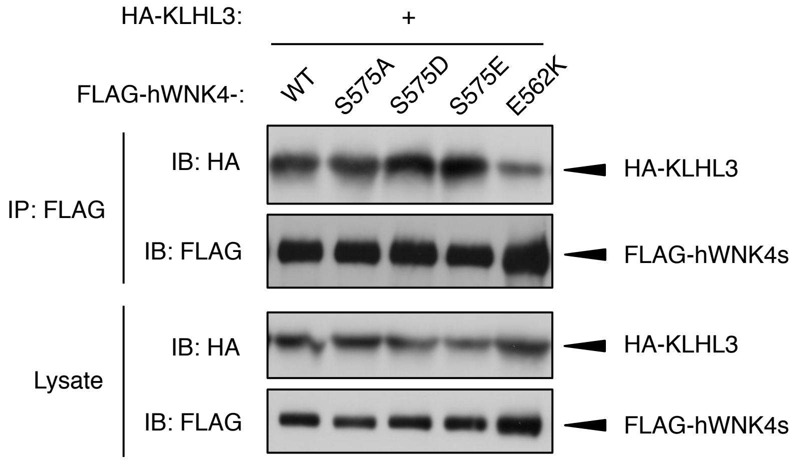

(b)

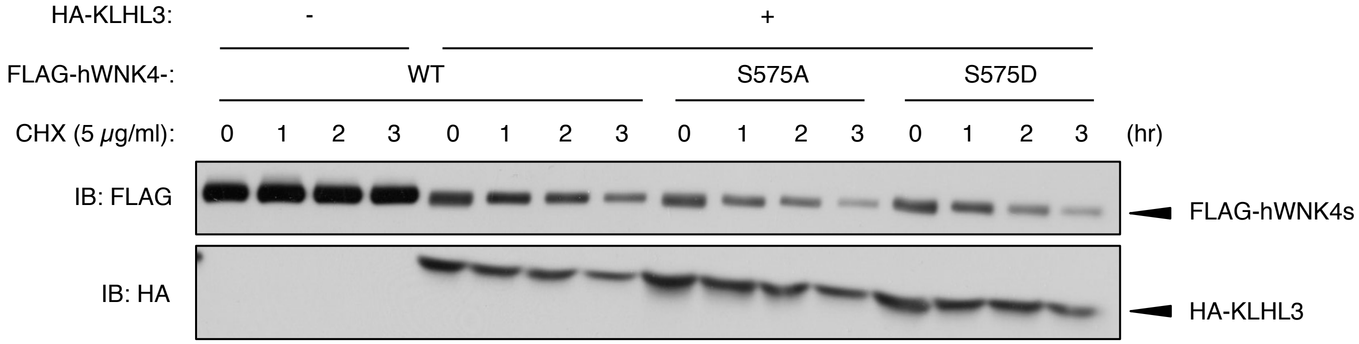

Supplementary Figure S5

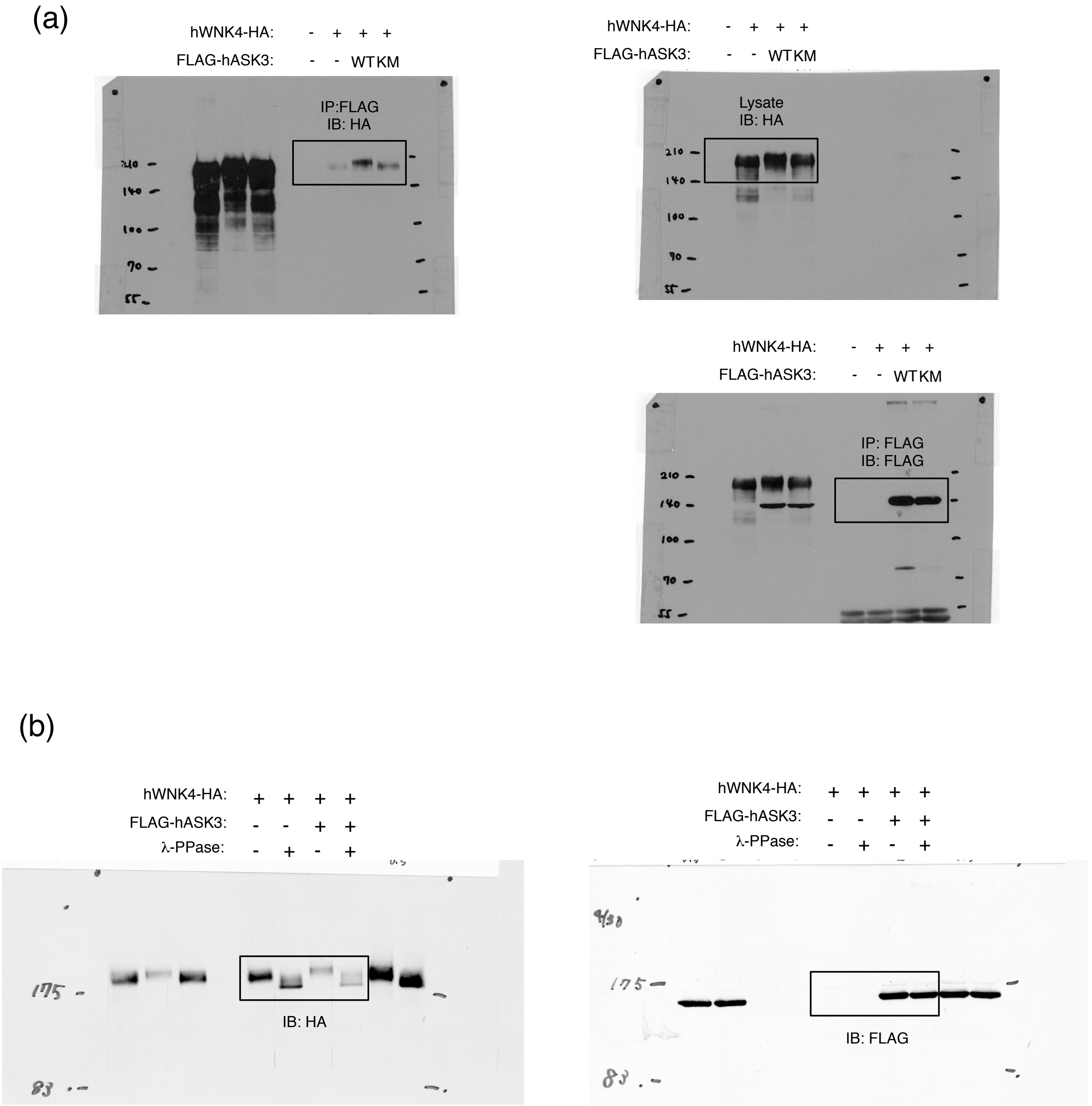

Supplementary Figure S6

(a)

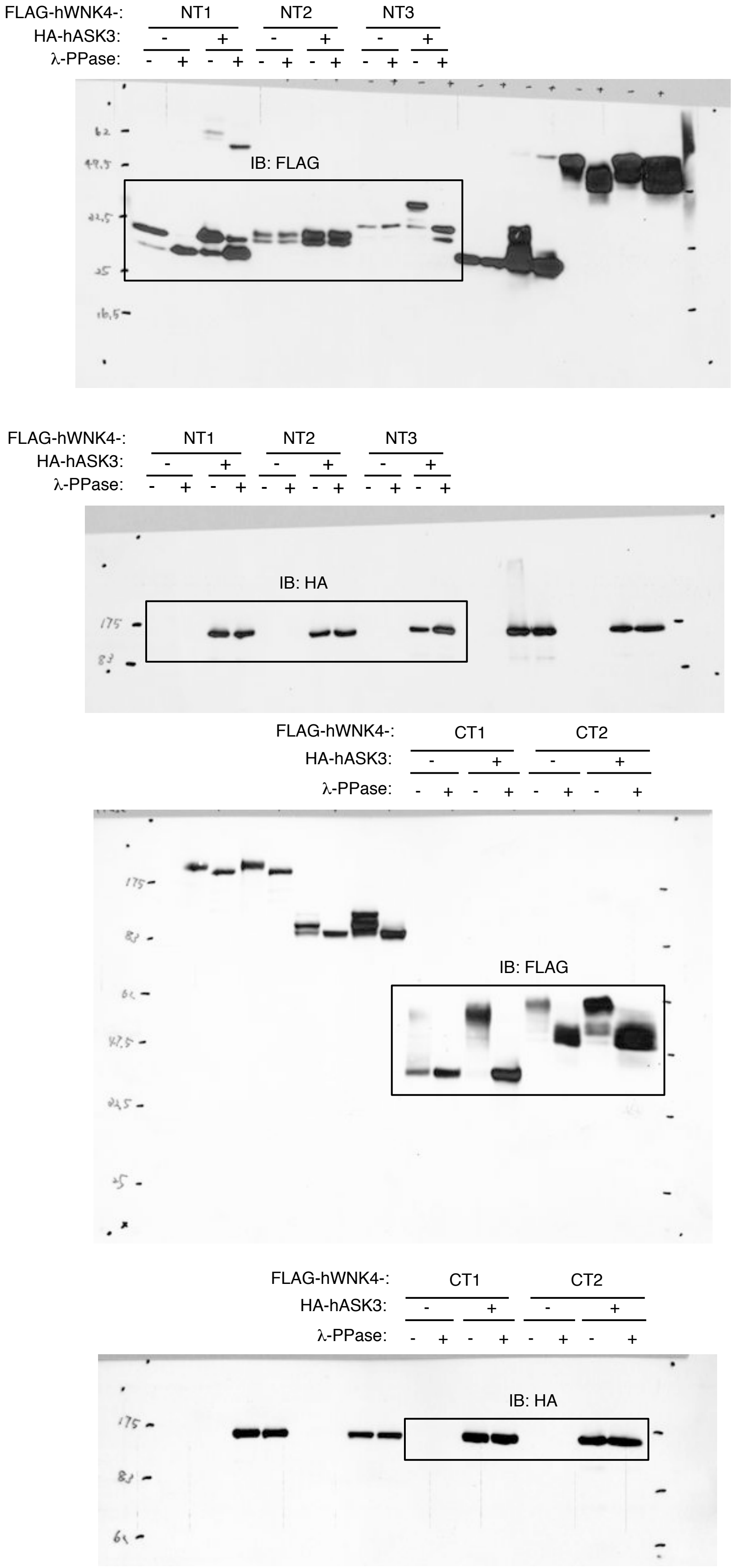

(b)

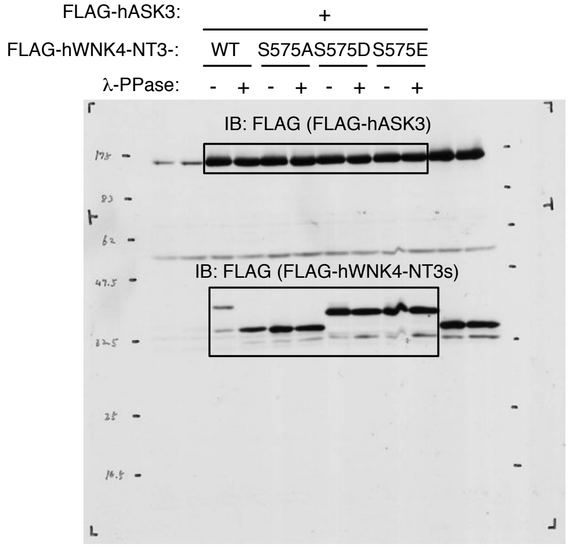

Supplementary Figure S7

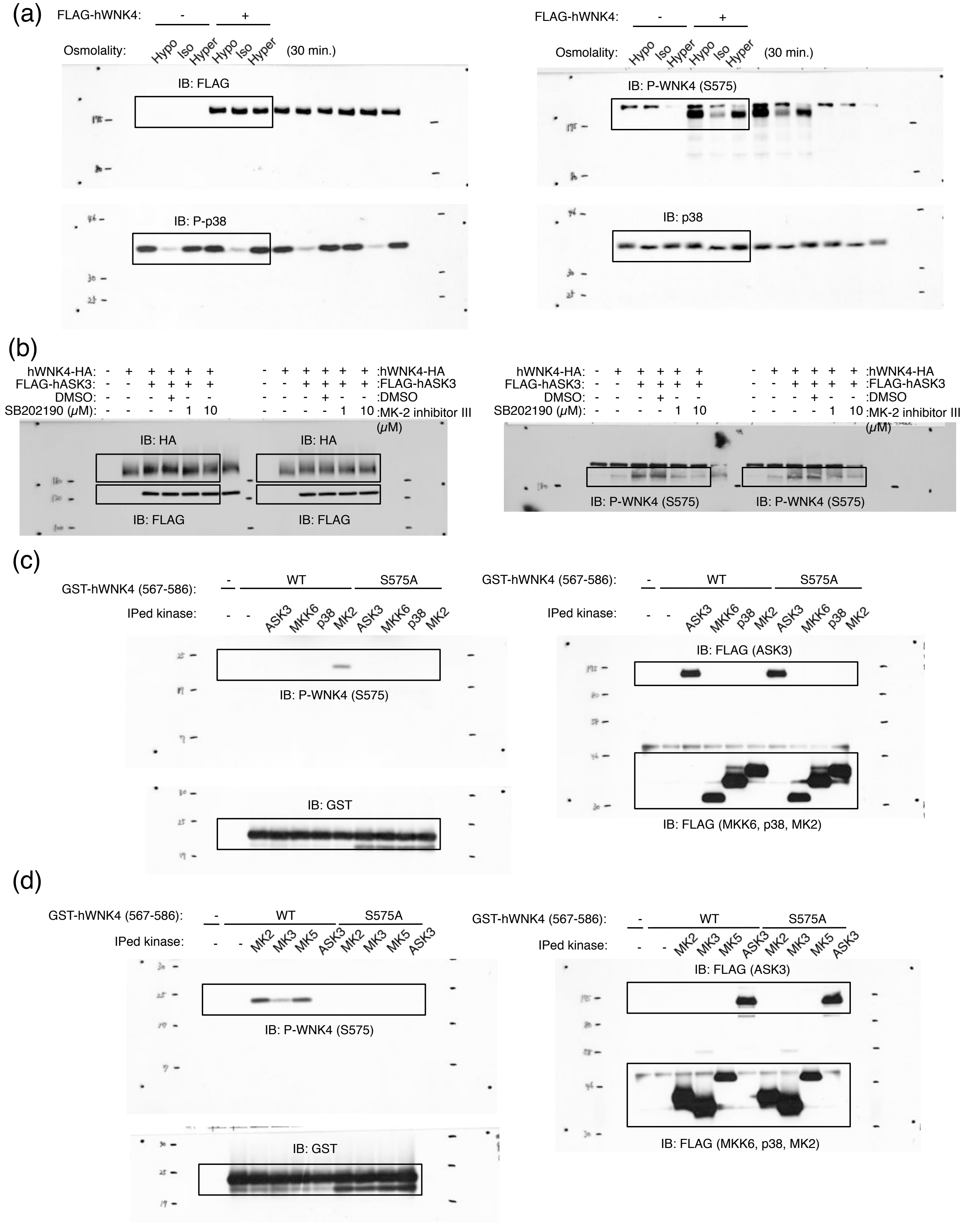

Supplementary Figure S8

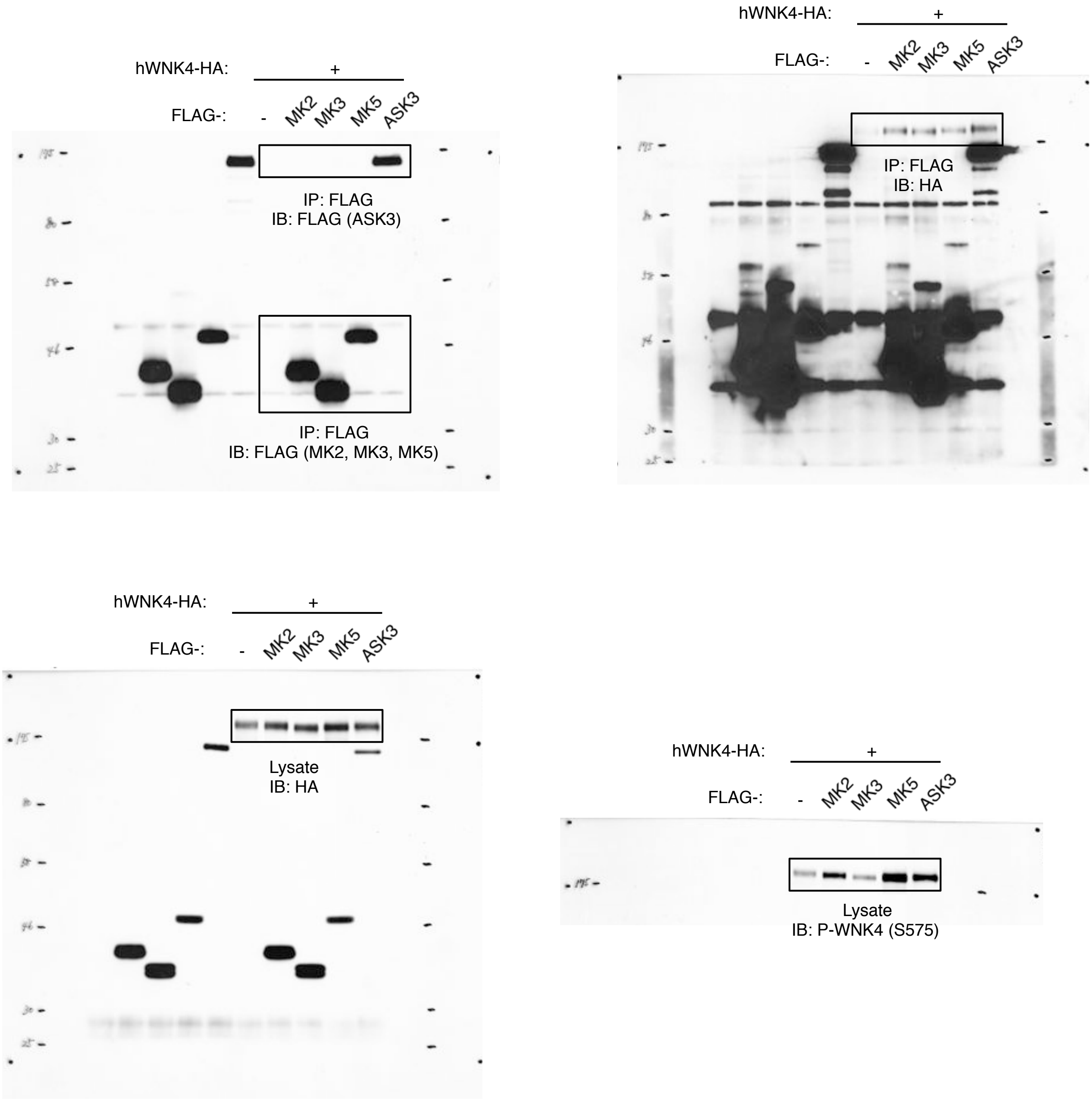

Supplementary Figure S9

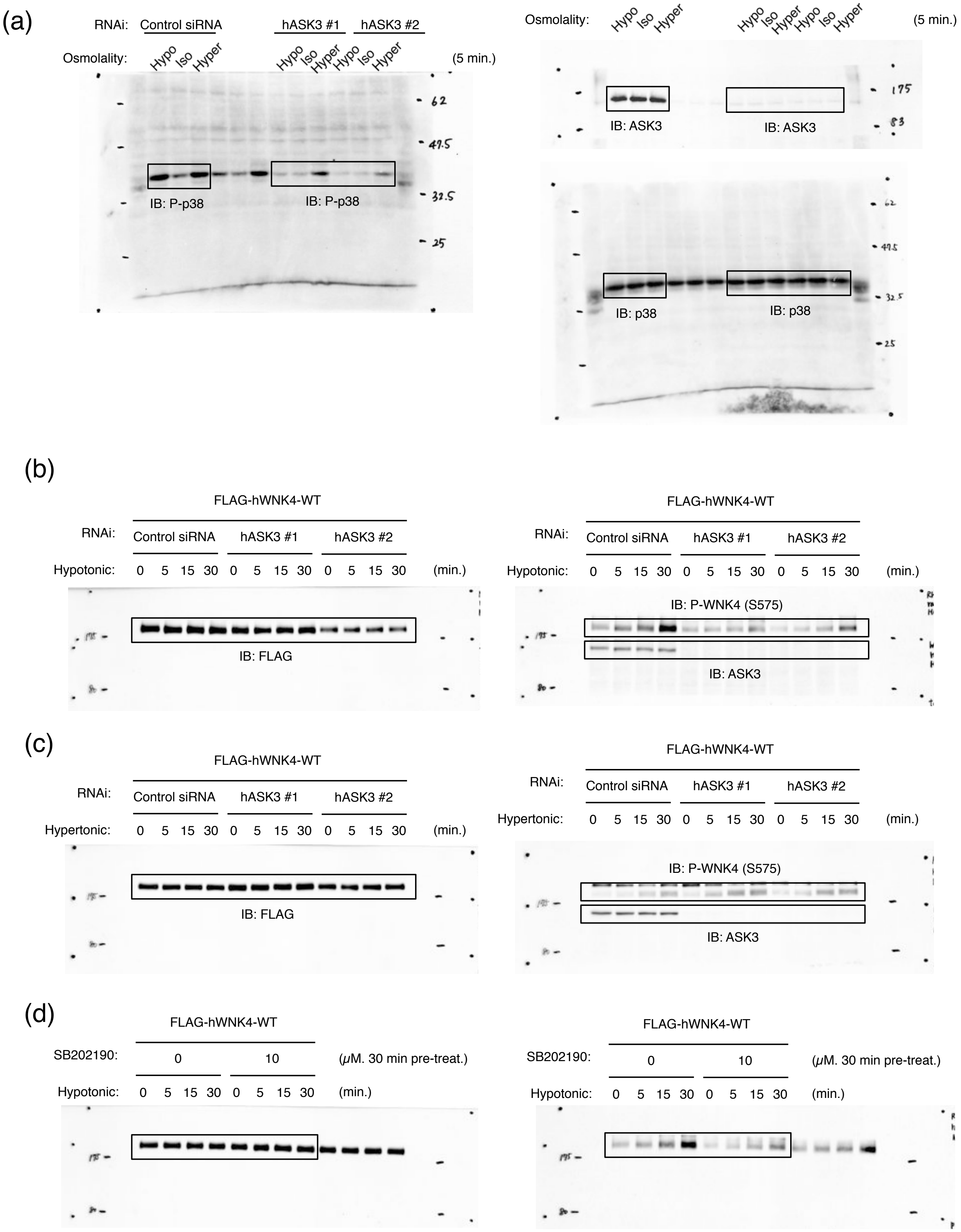

Supplementary Figure S10

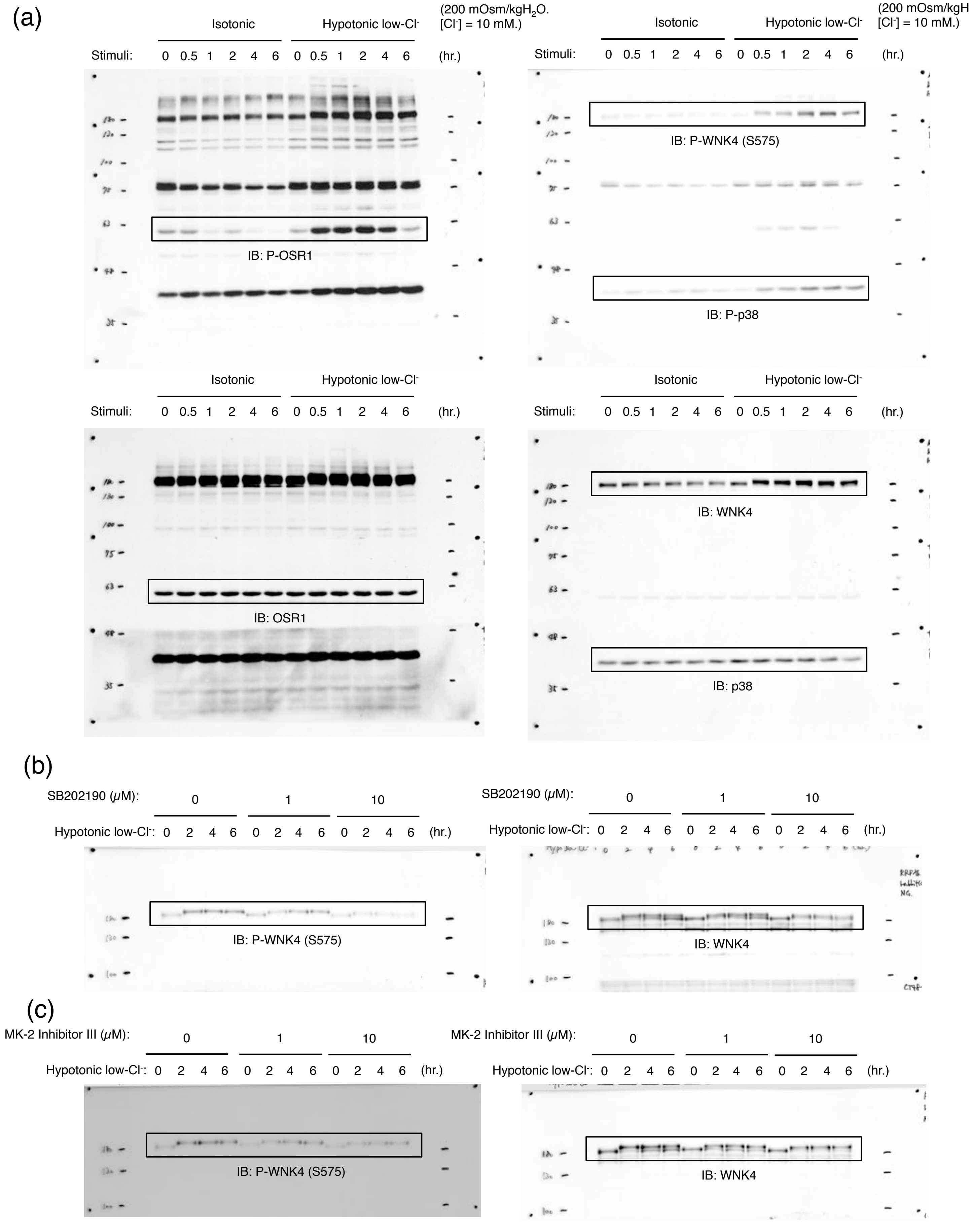

Supplementary Figure S11

(a)

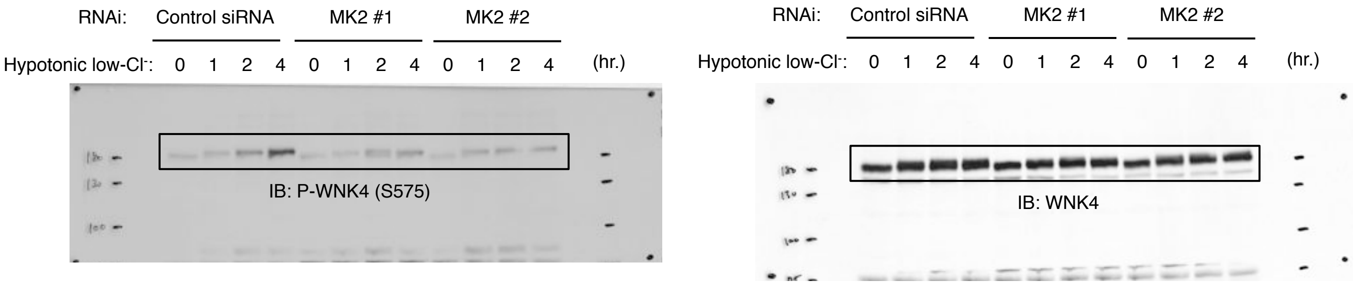

(b)

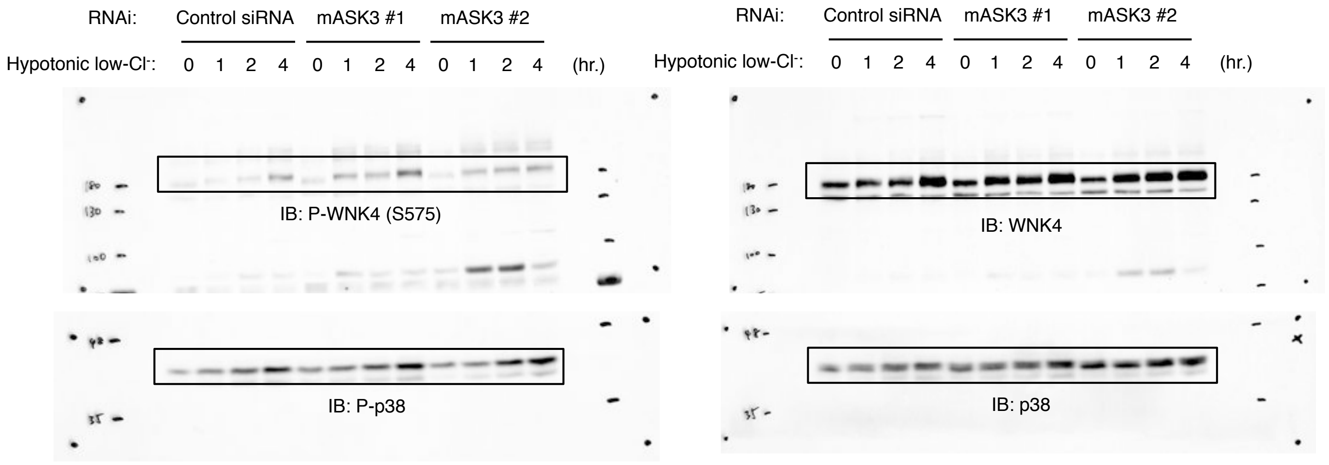

Supplementary Figure S12

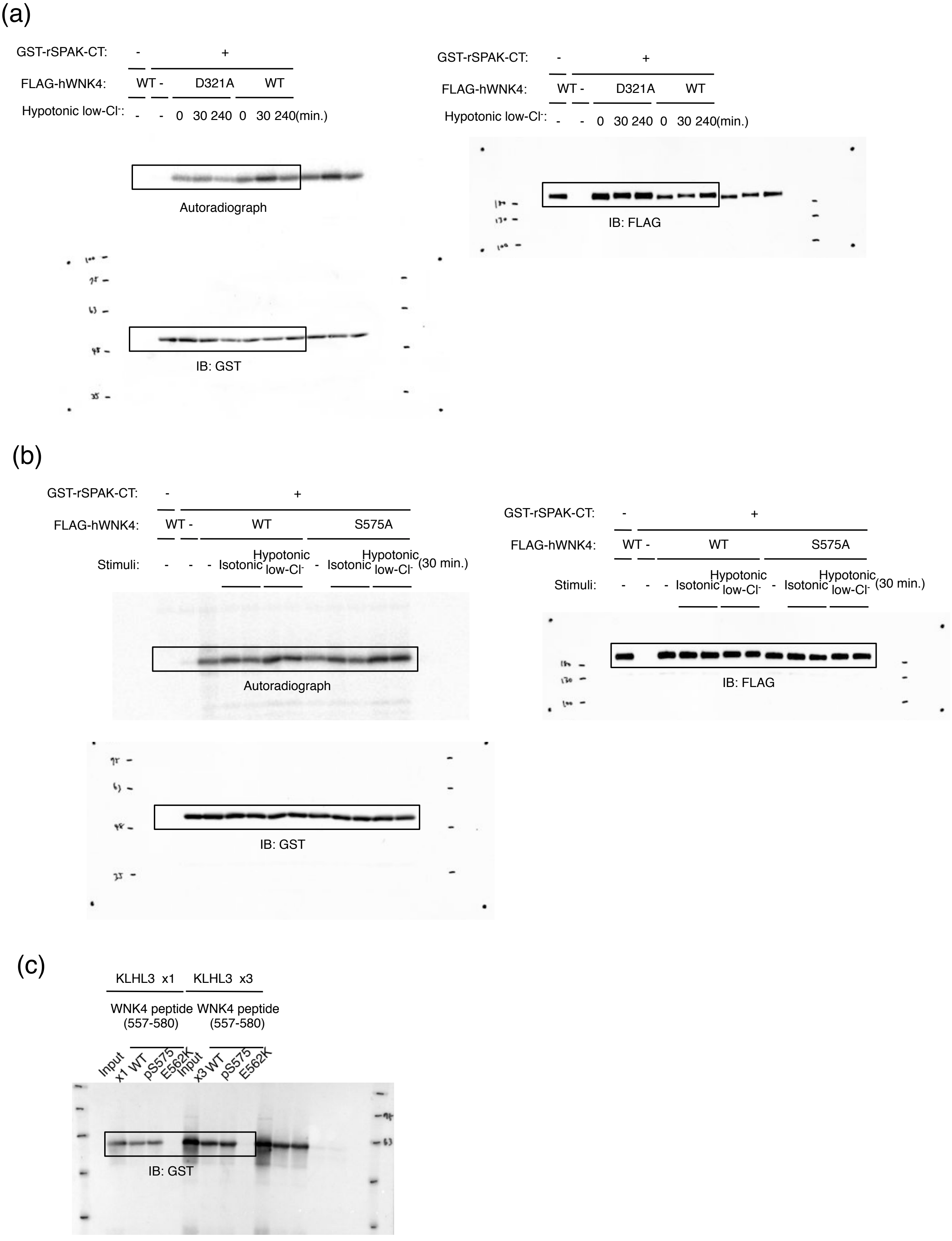

Supplement: Supplementary Information [file srep18710-s1.pdf]
